# A novel policy dialogue to build sustainable and resilient health systems: findings from PHSSR Portugal

**DOI:** 10.1186/s12961-025-01329-5

**Published:** 2025-05-19

**Authors:** Mónica D. Oliveira, Aida I. Tavares, Rafael Miranda, Rosário Trindade, Ana C. L. Vieira

**Affiliations:** 1 CEGIST, Instituto Superior Técnico, Universidade de Lisboa, Av. Rovisco Pais, 1049-001 Lisbon, Portugal; 2 iBB-Institute for Bioengineering and Biosciences and i4HB-Associate Laboratory Institute for Health and Bioeconomy, Instituto Superior Técnico, Universidade de Lisboa, Av. Rovisco Pais, 1049-001 Lisbon, Portugal; 3 ISEG, UL-Lisbon School of Economics and Management, University of Lisbon, Lisbon, Portugal; 4 CEISUC-Centre for Health Studies and Research, University of Coimbra, Coimbra, Portugal; 5 AstraZeneca Portugal, Oeiras, Portugal

**Keywords:** Policy dialogue, Health system sustainability, Health system resilience, Delphi, Dialogue mapping

## Abstract

**Background:**

In the aftermath of the COVID-19 pandemic, health policymakers have been reflecting upon sustainability and resilience issues in health systems worldwide. Promoting sustainability and resilience requires policy changes built upon evidence and on the views of health stakeholders and experts. This study aimed to engage health stakeholders in designing and discussing policy recommendations with a high potential to improve sustainability and resilience in the Portuguese healthcare system.

**Methods:**

As part of the Partnership for Health System Sustainability and Resilience initiative in Portugal (PHSSR-PT), this study proposes a novel policy dialogue that combines participatory methods—workshops and Web-Delphi processes—with content analysis tools—namely Dialogue Mapping—to promote agreement and help health stakeholders and experts to identify and discuss policy recommendations with high potential to improve health systems’ sustainability and resilience. Departing from the COVID-19 pandemic as a critical event and drawing on evidence and data, a group of health stakeholders and experts ideated and agreed upon high-value policy recommendations across seven domains: Governance, Financing, Workforce, Medicines and Technology, Service Delivery, Population Health and Environmental Sustainability.

**Results:**

40 top-level Portuguese health stakeholders and experts successfully collaborated to generate and discuss the benefits, risks and implementation issues of 69 policy recommendations, out of which 43 were selected as having gathered a high level of agreement on their potential to improve system sustainability and resilience. The adopted policy dialogue promoted high convergence. Many of these 43 recommendations were shown to entail interconnectedness with other policy recommendations.

**Conclusions:**

This study provides actionable insights to advance discussions on sustainability and resilience in Portugal. It shows that Governance and Population Health policy recommendations are critical to improve sustainability and resilience in several domains, and there is a high level of agreement on the need to adopt many recommendations, but questions remain about their implementation. The study also shows that new ways of engaging health stakeholders and experts can be adopted to promote dialogue, consensus and transparent discussion in policy processes.

**Supplementary Information:**

The online version contains supplementary material available at 10.1186/s12961-025-01329-5.

## Background

The coronavirus disease 2019 (COVID-19) pandemic challenged health systems worldwide [1], causing a multidimensional shock, as shown by overwhelmed hospitals and healthcare professionals, shortages of medical supplies, equipment and staff, increased demand for contact tracing and COVID-19 testing and extraordinary, unexpected, and unsustainable health related expenditures [2]. Portugal was not exempt from these challenges, which exposed vulnerabilities, particularly in the Portuguese National Health Service (NHS) [3]. The pandemic’s impacts included: excess mortality [4], highlighting the system’s inability to handle patient surges adequately; insufficient funding, increased debt [5] and high out-of-pocket expenditures [6], which strained financial sustainability, exacerbated existing inequalities [7] and undermined both the system’s equity and overall performance; burned-out healthcare workers [8], signaling unsustainable working conditions and staff shortages; and multiple other impacts, including long waiting lists [9], fragmented information systems [10] and poor mental health [11], exposing persistent inefficiencies and quality issues. Although it is recognized that the pandemic experience also presented learning opportunities (including for the Portuguese NHS [3, 12, 13]), policymakers worldwide increasingly recognize the importance of pursuing sustainability and resilience objectives in reforming healthcare systems [14, 15] and the need to consider evidence and data together with health stakeholders and experts engagement in the design of potentially valuable policies [16].

Within this context, the Partnership for Health System Sustainability and Resilience (PHSSR) emerged, assuming the motto that building sustainable and resilient health systems requires system-wide collaboration among governments, public and private providers, patient associations and other stakeholders [17]. The PHSSR initiative fosters global and cross-sector collaboration to strengthen health systems worldwide, making them better equipped to face future challenges and crises [18], being supported by AstraZeneca, KPMG, Royal Philips, the London School of Economics and Political Science, the World Economic Forum, the Center for Asia–Pacific Resilience and Innovation and the World Health Organization (WHO) Foundation. For implementing the initiative in multiple countries, the PHSSR created a general framework for analysing health systems’ sustainability and resilience, taking the view that ‘a sustainable health system (1) improves population health by (2) delivering the key functions of providing key services, generating resources, financing and stewardship, (3) incorporating principles of financial fairness, equity in access, responsiveness and efficiency of care, while (5) minimising its environmental impact, and (6) it does so continually by learning and adapting to changing contexts’ and ‘a resilient health system is able to (1) prevent, respond to, and manage the health system impact of, (2) and recover and learn from, (3) acute and chronic crises (including, but not limited to, pandemic threats, climate change and economic and technological shocks), (4) minimising their short- and long-term impacts on health, social and economic wellbeing’ [19].

Building on these definitions and on a review of multiple frameworks (which identified 80 attributes relevant to health system’s sustainability and resilience), the PHSSR framework defined seven critical domains for promoting sustainability and resilience in practice [19, 20]. Specifically, Governance encompasses the wide range of steering and rulemaking, exercised by governments and decision-makers to achieve health policy objectives. Financing refers to how health systems generate, pool and allocate financial resources and pay for services. Workforce examines how health systems plan for, train, recruit, reward and deploy their workforces and shape the conditions in which health professionals work. Medicines and Technology analyses how health systems make use of medicines and technologies in the delivery of services. Service Delivery relates to how health services are organized and delivered. Population Health looks at how health systems address the social determinants of health and demands of the population. Lastly, Environmental Sustainability refers to how health systems prevent and minimize their carbon footprint and the devastating effects of climate change.

The PHSSR framework acknowledges that sustainability and resilience definitions depict a false dichotomy and are operational constructs, with alternative frameworks being available (e.g. WHO framework for resilient and sustainable health systems [21] and others [22–24]). For instance, when comparing the PHSSR to the WHO framework [21], both were developed in response to the pandemic and aim to strengthen health systems’ shock response and long-term performance through core elements such as Governance, Financing, Workforce and Service Delivery, as well as emphasize preparedness, adaptation, recovery and evidence-based policy. However, while the WHO framework focuses on the European Region and is more prescriptive, offering defined performance indicators and metrics, PHSSR was designed to be more flexible, to use qualitative and stakeholder-driven policy dialogues and adding domains such as environmental sustainability and population health.

The PHSSR initiative spanned across multiple countries, with this study reporting its implementation experience in Portugal (henceforth PHSSR-PT). Scientific literature on Portugal has not comprehensively addressed sustainability and resilience nor performed structured policy dialogues, leaving a gap in published studies. Before the pandemic, Conill et al. [25] discussed social determinants of health and financial sustainability, Nunes and Ferreira [26] analysed the NHS response to citizens’ needs and Russo et al. [27] evaluated the resilience of health professionals and the services market during the 2010–2015 crisis. The Portuguese Gulbenkian Foundation created a commission in 2012, led by Nigel Crisp [28], which implemented a policy dialogue involving over 50 individuals in four working groups to develop 20 recommendations on Governance, Social Determinants, Care Delivery and Financial Sustainability. After the pandemic, some studies explored sustainability and resilience aspects—Kuhlmann et al. [29] briefly addressed healthcare workforce resilience and sustainability, Willems et al. [30] assessed primary care crisis preparedness and Kuhlmann et al. [31] issued recommendations on the primary healthcare workforce. A recent report [32] from the Portuguese Health Foundation Observatory reflected on expectations versus the reality of the NHS.

Bearing in mind the need to reflect upon sustainability and resilience in the pandemic aftermath, the primary objective of PHSSR-PT was to generate evidence-based knowledge and engage health stakeholders in a reflection on how to promote sustainability and resilience in the Portuguese health system (policy-oriented aim) [33]. To support this, a secondary (methodological-oriented) aim was to design a novel fit for purpose policy dialogue.

The need for a novel policy dialogue arose from reviewing the literature and analysing the requirements for successful dialogue in the Portuguese context. General literature shows that successful policy dialogues require the use of comprehensive approaches [34] aligned with the pursuit of policy objectives [35]. Previous reported policy dialogues focus mostly on sector-specific discussions, such as health workforce issues [36] or national health financing strategies [37]. Other studies [38, 39] emphasize the value of multi-stakeholder engagement but focus primarily on conceptual discussions rather than on applied methodologies for structuring these dialogues, not providing a structured, general framework for engaging diverse stakeholders in a systematic and evidence-based manner.

Additionally, specific requirements were identified as relevant for a successful PHSSR-PT policy dialogue. First, it should facilitate the identification of policy recommendations with significant potential to enhance the country’s sustainability and resilience [40]. Second, it should embrace differing opinions, foster consensus and engage diverse stakeholders to ensure varied perspectives [41, 42]. Third, it should guarantee that participants are well-informed and knowledgeable, with access to up-to-date, robust evidence [43]. Finally, there is the call for support tools to enable an understanding about policies’ benefits, risks and costs, ensuring policies provide value for money and align with sustainability and resilience goals [44].

Hence, within PHSSR-PT, we introduced a novel (structured, transparent and operational) policy dialogue to foster open discussion and agreement among Portuguese health stakeholders and experts on policy recommendations with a high potential to improve sustainability and resilience, which can be applied to other policy settings.

The remainder of the article outlines the individual stages (and employed methods) of the policy dialogue and the results from ideation, refinement and selection of the most relevant policy recommendations for improving health system sustainability and resilience in Portugal. Finally, we draw insights on the policy recommendations, on the policy dialogue and on study limitations.

## Methods

### Policy dialogue design

**Fig. 1:**
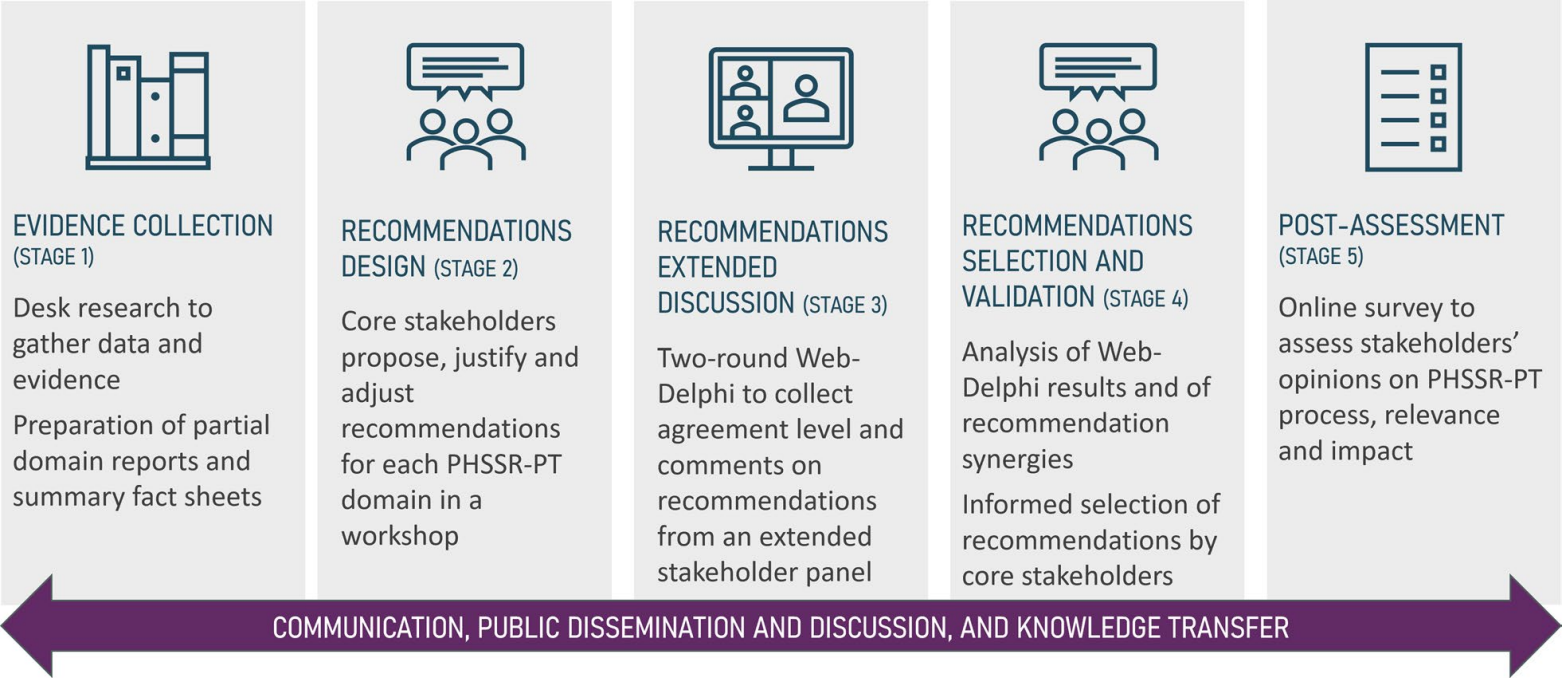
Five-stage policy dialogue approach adopted

The fit for purpose dialogue, designed to engage health stakeholders and experts in ideating, discussing and selecting policy recommendations (while promoting consensus), follows the process detailed in Fig. 1 and is structured in the five stages (described below), concerning evidence collection and synthesis (stage 1), design of policy recommendations (stage 2), extended discussion of policy recommendations (stage 3), policy recommendations’ selection and validation (stage 4) and, finally, a post-assessment of the process (stage 5).

Conceptually, this policy dialogue follows a collaborative value modelling approach (as set in Vieira et al. [44]), combining social—surveys, workshops and Web-Delphi [45]—with technical processes, including narrative reviews of studies and dialogue mapping [46]. Distinct social methods are combined to ensure rigour and inclusiveness in the policy dialogue process. Namely, group-based approaches are favoured to individual-based approaches (such as interviews) on grounds that they inherently promote an interactive and dynamic exchange of ideas between the stakeholders [31]. The survey format in stage 2 ensures that initial policy recommendations are well-structured and grounded in individual expert input before group discussion, promoting freedom for participants expressing their views without external pressure, and the workshop in stage 2 enables clarifying the rationale and description of policy recommendations. The Delphi method (stage 3) is selected due to its ability to engage a large number of stakeholders and systematically promote participants’ agreement while preserving anonymity, which minimizes the influence of dominant voices and encourages diverse perspectives [45]. This is particularly important given that health stakeholders and experts may have differing institutional or political positions that can influence open discourse (this aspect lead to the Delphi being preferred to focus groups and expert panel formats). The iterative nature of the Delphi process also enables stakeholders and experts to refine their opinions based on anonymized feedback from their peers. The final workshop (stage 4) provides stakeholders with a space for individual reflection, followed by a group discussion, enabling them to share perspectives, debate policy recommendations and collaboratively refine and select recommendations. The post assessment stage (stage 5), operationalized through a structured survey, promotes a final individual reflection both at the level of the design and results of the process.

This study was approved by the Ethics Committee of Instituto Superior Técnico, University of Lisbon (ref. no. 4/2022 (CE-IST), 4 April 2022).

### Stage 1: evidence collection

Following the PHSSR framework and corresponding guidelines [19], a set of indicative (but not constraining) questions per PHSSR domain were used to collect and digest evidence (e.g. ‘Are there specific policies or programmes in place to reduce wasteful expenditure in the health system?’ [Service Delivery] and ‘Who are the key actors in the governance of the health system and what are their areas of responsibility?’ [Governance]) and to make a critical assessment on sustainability and resilience in the Portuguese context. The PHSSR England report [47] was also provided as a good example of report. Accordingly, desk research using recent data and relevant publications was used to prepare a narrative report for each PHSSR domain and then strengths and weaknesses from the viewpoint of health system sustainability and resilience were synthesized (for each domain).

To enable an effective communication of these analyses in the following stages of the policy dialogue [48], seven summary ‘Fact and Evidence’ sheets were produced. Each ‘Fact and Evidence’ sheet comprised three sections: (1) a synthesis of key findings on the domain’s strengths and weaknesses regarding sustainability and resilience; (2) a detailed presentation of key domain evidence and data, including subsections on relevant domain topics, e.g. for Service Delivery, included subsections are: efficiency of care related aspects, quality of care, coordination of care and new care models, distribution and access to health services, programmes for prevention and for chronic diseases and COVID-19 pandemic; and (3) a brief section on the methods used to produce the sheet.

### Stage 2: recommendations’ design

A core group of renowned Portuguese health stakeholders and experts was invited to design and select public policy recommendations to enhance the sustainability and resilience of the Portuguese health system (stakeholders and experts to be engaged in stages 2–5). Group members were selected to ensure expertise across all PHSSR domains—with key competencies mapped by domain to ensure the group's general coverage of related topics—and a diversity in backgrounds, professional experience (current and past), sectors (working in public/private/social sector organizations, academia, patient associations and industry) and political perspectives.

During the recommendations’ design stage, core group members were asked to individually propose policy recommendations: ‘Taking into account the data and evidence provided on the Portuguese health system [i.e. the ‘Fact and Evidence’ sheets], propose (for each of the following domains) the policy recommendation that you believe has the greatest potential to improve sustainability and resilience’. Participants submitted their proposals and respective rationale electronically using a template (Supplementary File 1). It was not mandatory to suggest recommendations for every domain.

The responses were collated into a structured and standardized document, which was presented to the core group in a workshop setting. During the workshop, participants were invited to discuss and review the names and rationale of the proposed policy recommendations to ensure a shared and clear understanding of their content and framing. Conflicting recommendations were allowed to respect the different views and similar or overlapping recommendations were merged. After the workshop, participants received a workshop report with all policy recommendations and were given four working days to suggest improvements to their framing.

### Stage 3: recommendations’ extended discussion

The policy recommendations (and their rationale) from stage 2, together with the ‘Fact and Evidence’ sheets from stage 1, served as input for a two-round Web-Delphi process. In addition to members of the core group, an extended panel of health stakeholders and experts was invited to (1) indicate their level of agreement on the extent to which each policy recommendation had a strong potential to improve the sustainability and resilience of the Portuguese health system and (2) provide anonymous comments to justify their answers and/or comment on the proposed recommendations. Members of the core group and of the PHSSR-PT research team identified health stakeholders and experts to ensure a broad range of expertise across PHSSR domains—performing analyses similar to the ones carried out on the composition of the core group, the key competencies of the members invited to the panel were mapped to ensure comprehensive coverage.

**Fig. 2:**
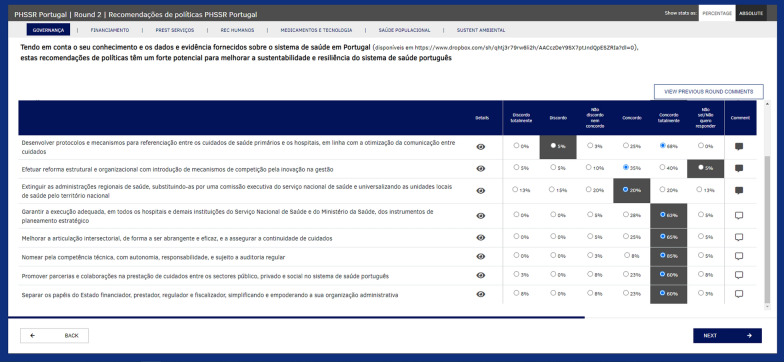
Web-Delphi process on the Welphi platform, displaying recommendations from the Governance PHSSR domain (in Portuguese) during round 2. The screen shows the proposition: ‘Taking into account your knowledge, evidence and data about the Portuguese health system, these policy recommendations have strong potential to improve system sustainability and resilience’ (bold), a five-level Likert scale (columns on the right) and a list of policy recommendations (left) with an eye icon for their rationales

The Web-Delphi process was conducted in two rounds on the Welphi platform (http://www.welphi.com/, see example of a round 2 screen in Fig. 2). In both rounds, experts and stakeholders evaluated recommendations on a five-level Likert scale, ranging from ‘totally disagree’ to ‘totally agree’, with an additional ‘Don’t know/don’t want to answer’ option being available. Participants’ action was guided through the list of policy recommendations and their justifications and by accessing the information available in the ‘Fact and Evidence’ sheets. After giving their initial agreement levels in round 1, participants could review their answers in round 2 while taking into consideration their peers’ answers and comments presented in an anonymized way.

The Delphi results were analysed and utilized through two approaches. First, a statistical synthesis of the final Delphi round was prepared, where the following net agreement system was applied:

- as defined in the left side of Eq. 1, where TA signifies ‘totally agree’, A is ‘agree’, TD is ‘Totally disagree’ and D is ‘disagree’), and a discordance factor of 2 was adopted, by which a double weight is assigned to dissenting votes compared with agreeing ones;
- it was indicatively defined that a policy recommendation should be considered when gathering a qualified majority for the net agreement minus twice the disagreement [49]:
  1 $$ \left[ {TA\left( \% \right) + A\left( \% \right)} \right] - 2\left[ {TD\left( \% \right) + D\left( \% \right)} \right] \ge 75\% , $$

Second, following stages 2 and 3, the input provided by stakeholders and experts for each policy recommendation—recommendations’ rationales and Web-Delphi comments—was synthesized using dialogue mapping. Dialogue mapping is a problem structuring method that enables the systematic organization and visualization of complex discussions [46, 50], and, in this specific context, was used to map expected benefits, risks, costs, implementation barriers and other issues (including lack of clarity in the recommendation, if recalled by participants).

### Stage 4: recommendations’ selection and validation

A report containing all recommendations, statistical results and comments of the Delphi process, together with Dialogue Maps, was sent to the core group previous to a second workshop, to which core group members were invited to: collectively analyse and discuss the Web-Delphi results and the net agreement rule, select policy recommendations and work upon the text (name and rationale) of the selected (final) policy recommendations, considering the views of the extended panel. After the workshop, participants received a workshop report and were invited to make final adjustments or validate the final policy recommendations.

Furthermore, the research team also examined potential interconnections among the final recommendations by employing a pragmatic adaptation of Friend and Hickling’s application [51] of the Analysis of Interconnected Decision Areas (AIDA) technique [52]. Generally, AIDA enables the representation of links between various ‘decision areas’, each comprising multiple strategic options, then assesses their compatibility to create a portfolio of interconnected strategic options, addressing a specific problem [53]. This technique [51] serves as an informal ‘intermediate technology’ that facilitates communication within decision-making groups by graphically expressing complex problem structures. Here, a simplified approach was adopted by analysing the extent to which the rationale and comments for a given policy recommendation were recalled for the implementation of other policy recommendations. Totals were then calculated by PHSSR domain to identify the most interconnected domains, providing a networked view of the relationships between recommendations within and across domains. The use of a Sankey diagram was chosen to synthesize the results [54] (in the format later presented in Fig. 5).

### Stage 5: post-assessment

After disseminating the study’s analysis, conclusions and recommendations, a post-assessment exercise was performed. It entailed a semi-structured online questionnaire to members of the core group for them to assess the timeliness, relevance and impact of the project and policy dialogue for Portuguese health policymaking. The questionnaire was individual and anonymous and entailed two parts. The first part included 16 statements across five thematic areas related to the participatory process: information base, proposed recommendations, Web-Delphi process, integrated approach and PHSSR-PT initiative. Participants rated their agreement on a five-level Likert scale from ‘totally disagree’ to ‘totally agree’, with an ‘I don’t know/I don’t want to answer’ option. The specific statements are later presented in Table 4. The second part had open response fields for participants to (1) list the three most relevant projects for the Portuguese health system (if not PHSSR-PT), (2) indicate three key initiatives for implementing the study recommendations and (3) provide any other comments or suggestions on the PHSSR-PT initiative.

### Communication, public dissemination and discussion and knowledge transfer

Targeted initiatives to ensure effective communication and dissemination of results were discussed from the outset of the project. A communication strategy was developed to promote a broad public discussion on health system sustainability and resilience and to sustain stakeholder engagement, including with government representatives, the Ministry of Health (MoH), political parties, NHS bodies and civil society organizations; additionally, the communication strategy was set to promote the policy dialogue, raise awareness and secure support for recommendations’ implementation. Key planned activities included national press releases, evidence dissemination, media coverage (e.g. newspapers, radio and TV) and high-profile events. Further efforts involved public forums and roundtables to discuss recommendations.

## Results

### Stage 1: evidence collection

Seven detailed partial (narrative) reports (one for each PHSSR domain, available as sections of [33]) and ‘Fact and Evidence’ sheets (available in Supplementary File 2) were produced for the assessment of sustainability and resilience in the Portuguese health system. Table 1 provides a synthesis of key findings and references for the assessment in each of the seven sustainability and resilience domains. A full list of references can be found in the full report [33].

**Table 1:** Sustainability and resilience—summary of key findings by PHSSR domains presented in [33]

Domain (key references)	Sustainability		Resilience	
	Strengths	Weaknesses	Strengths	Weaknesses
Governance [55–59]	Clear governance enables decision-making	Low accountability Underdeveloped policy evaluation Fragmented information systems	A command-and-control system enabling rapid decision-making, and inter-sectoral cooperation through the pandemic	Political resoluteness Often reactive rather than proactive pandemic responses Weak contingent planning
Financing [59–63]	Risk pooling and universal coverage	Excessive out-of-pocket Systematic budget deficits driving retrospective financing	New financial policy measures were adopted to address the pandemic	Reactive spending under the pandemic Lack of needs consideration for financial reforms
Workforce [64–68]	Strengthening of human resources after the pandemic	Lack of financial incentives and pay Low professional satisfaction No strategic planning High shortage of doctors in some medical specialities	High adaptability during the pandemic Redeployment and sharing of human resources	Unsustainable extra costs during the pandemic Mental health issues
Medicines and Technology [55, 69–71]	Effective cost containment measures on pharmaceutical expenditure Established HTA	Delays in access to therapeutic innovation Low level of R&D Low evaluation of technologies Financial pressure from hospital medicines	Adaptability and increase in telemedicine use Effective COVID-19 vaccination strategy and delivery based on telephone consultations	Financial risk related to health technology innovation Most telemedicine is based on telephone consultations
Service Delivery [64, 65, 72–75]	Extensive primary care network Emergence of more integrated delivery models	Hospital-centric response to inadequacies in primary care Geographic inequities in healthcare Low focus on health promotion and prevention	A sharp increase in telehealth consultations during the pandemic Flexible private supply complementary to the NHS	Heavy decline in consultations during the pandemic Increase in surgical waiting times

	Sustainability and resilience	
	Strengths	Weaknesses
Population Health [7, 64, 65, 76]	Good performance regarding infant mortality and preventable and treatable mortality Low barriers to healthcare Widespread health programmes in schools Overall high immunization in the population	High burden of disease and low quality of life among older people High inequalities and inequities in health determinants Low health in lifestyle-related indicators
Environmental Sustainability [77–80]	Ongoing initiatives and legal framework supporting environmental sustainability Some initiatives for environmental sustainability led by the Ministry of Health in hospitals reaching maturity	Environmental sustainability measures are most centred in hospitals and focused on energy, water and waste Lack of policy actions Lack of assessment of policy benefits and costs

*HTA* Health technology assessment, *NHS* National Health Service, *R&D* Research and development

Analysis of sustainability and resilience in the PHSSR domains (as entailed in the ‘Fact and Evidence’ sheets and in Table 1) shows that the Portuguese NHS is under severe strain, with some problems being amplified with the pandemic. Portugal’s health Governance reflects limited inter-sectoral collaboration, with weak connections between hospitals and primary care and poor integration between independent health institutions. Although some public entities are responsible for promoting transparency and accountability in the NHS, managerial accountability remains low, particularly regarding budgetary deficits and overspending. In terms of Financing, the NHS is primarily tax-funded, ensuring universal coverage, yet it faces chronic budget deficits and high out-of-pocket spending (31% in 2019). Capital injections aimed at reducing public debt coexist with persistent issues such as delayed payments to suppliers, financial pressure from an ageing population and rising costs of innovative care. These challenges highlight the need for structural reforms, such as adopting multi-annual budgeting.

Portugal’s health Workforce has expanded, with increasing densities of doctors (above the EU average) and nurses (below the EU average), though doctor numbers may be overestimated due to statistical counting procedures. Despite this growth, shortages persist in some specialities, and there is a lack of human resource planning and retention strategies for NHS doctors. Formal long-term care workers have increased, but informal caregivers—vital due to ageing and low long-term care spending—remain largely undocumented. Regarding medicines and Technology, Portugal employs cost-effectiveness and budget impact assessments from the NHS perspective, with growing patient involvement. However, there is no apparent use of evidence-based medicine, and guidelines for medical device evaluation are underdeveloped. Centralized procurement exists for high-cost medicines, but funding for innovations is insufficient. While generics and biosimilars are growing, delays in drug approvals indicate ongoing difficulties balancing innovation and cost sustainability.

Service Delivery challenges include low hospital admission rates but increasing readmissions and high hospital infection rates. Intensive care units’ capacities are limited when internationally compared, leading to high occupancy and longer stays. Despite efficiency initiatives such as SNS24, the Coronary Green Way and MySNS, electronic health records are not fully implemented, and elective surgical waiting times remain high. Population Health reveals deep inequalities. High out-of-pocket costs and unmet care needs affect low-income and elderly populations. Geographic disparities in doctor distribution, energy poverty, higher mortality in interior regions and rapid ageing contribute to worsening social determinants of health. On Environmental Sustainability, although efforts have been made to reduce emissions and resource consumption, implementation is uneven. Greater central-level decision-making and resource allocation are needed to systematically embed environmental sustainability into health policy.

### Stage 2: recommendations’ design

Eleven Portuguese health stakeholders and experts composed the core group. By background, the group included three medical doctors, five economists/managers, two legal experts and one pharmacist, and by current professional roles, it included five health economists/researchers, two members working in hospitals or other provider organizations, two members from the pharmaceutical/medical devices industry, one member associated with the Ministry of Health or its agencies and one was a patients’ association representative. Notably, the group included a former Minister of Health, two former policymakers, a current policymaker and three members with clinical leadership and/or care delivery experience. Current or former policymakers were associated with distinct political parties. Females made up 37% of the group, and 37% of group members had private sector affiliations. By PHSSR domain, 8 members of the core group had key competencies in Governance, 8 in Financing, 8 in Workforce, 10 in Medicines and Technology, 9 in Service Delivery, 8 in Population Health and 3 in Environmental Sustainability, which ensured comprehensive knowledge and experience in all PHSSR domains.

Core group members submitted, via email (using the template), 70 policy recommendations. On 29 June 2022, a hybrid workshop (in-person and online) was held. The workshop resulted in 69 reviewed recommendations across the seven PHSSR domains, with their full details and rationale available in Supplementary File 3 and their names in Table 3. Eleven recommendations emerged to improve sustainability and resilience in the Governance domain, focusing on enhancing autonomy, reducing fragmentation and improving leadership and recognition of merit. Nine recommendations emerged concerning Financing, mostly related to delays in provider payments, stability in funding and promoting efficiency in insurance models. Eight recommendations emerged for Workforce, targeting staff shortages, low pay and inadequate human resource planning. For Medicines and Technology, 11 recommendations targeted the slow adoption of innovative treatments, poor procurement practices and outdated medical equipment. A total of 12 recommendations emerged for Service Delivery, addressing long wait times, weak integration across healthcare levels and inefficient digital systems, the same number as for Population Health, in which recommendations targeted poor preventive care, weak health literacy and challenges in elderly care. Finally, six recommendations were proposed for Environmental Sustainability, focusing on high energy consumption, unsustainable procurement and outdated hospital equipment, in line with promoting greener healthcare policies.

### Stage 3: recommendations’ extended discussion

A total of 70 experts, including 11 core group members and 59 additional high-level Portuguese health stakeholders and experts, were invited to participate in the Web-Delphi process. By PHSSR domain, 56 invitees had key competencies in Governance, 50 in Financing, 41 in Workforce, 37 in Medicines and Technology, 53 in Service Delivery, 54 in Population Health and 30 in Environmental Sustainability. Of those invited, 46 (66%) expressed willingness and availability to participate. The first Delphi round (starting 15 July 2022) was completed by 40 participants (87% acceptance rate), with 37 (7.5% dropout rate) completing the second round (ending August 29, 2022). Table 2 provides details on the self-reported characteristics (i.e. age and gender distribution, work sector and stakeholder group) of the participants who completed the second Delphi round.

**Table 2:** Self-reported characteristics of participants who completed the second round of the Web-Delphi process (*N* = 37)

Characteristic		Count	Share (%)
Age group	[30–49]	10	27.0
	[50–59]	13	35.1
	60 or more	14	37.8
Gender group	Female	12	32.4
	Male	25	67.6
	Other	0	0
Professional sector of main activity	Public	20	54.1
	Private	9	24.3
	Social	4	10.8
	Public and private	3	8.1
	Private and social	1	2.7
Professional position or function	Academic	8	21.6
	Decision-maker/ex-decision maker	10	27.0
	Health care or service provider	3	8.1
	Health professional	9	24.3
	Industry	4	10.8
	Patient/patient association	3	8.1

The final round’s distribution of panel responses is presented in Table 3, including net agreement percentages and whether recommendations met the (indicative) 75% selection threshold (net agreement in bold).

**Table 3:**
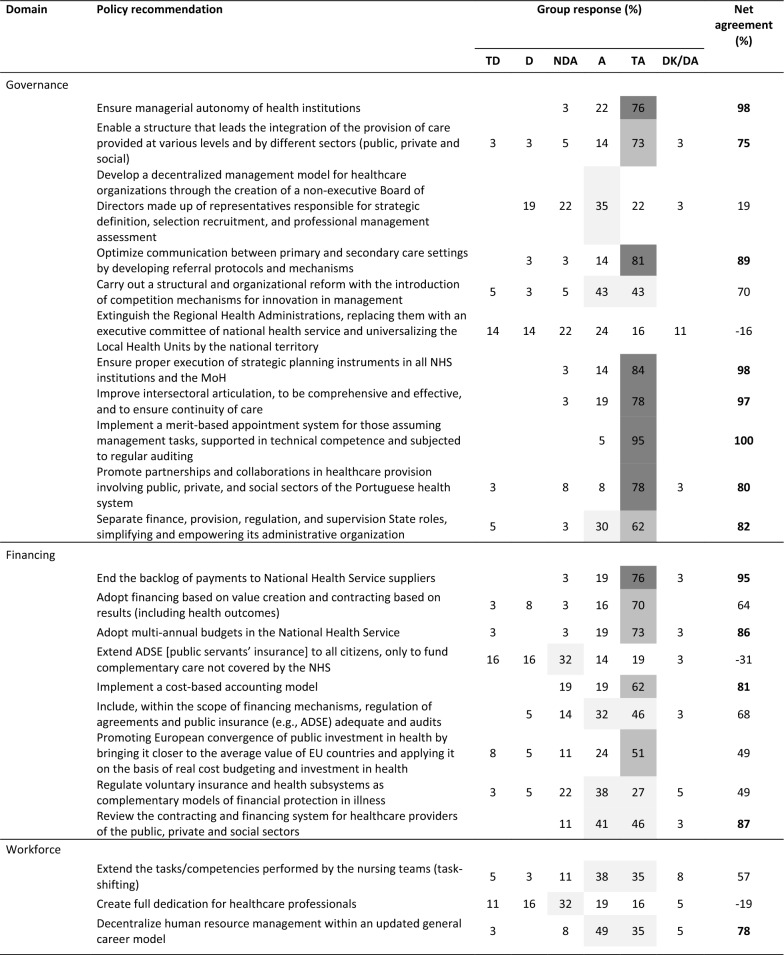

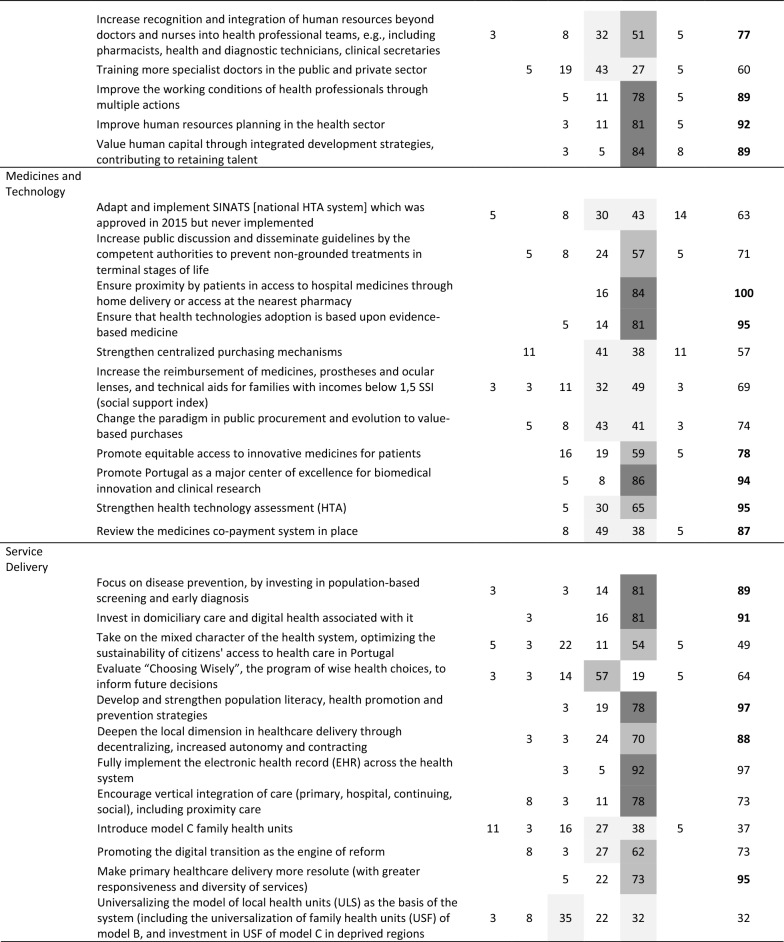

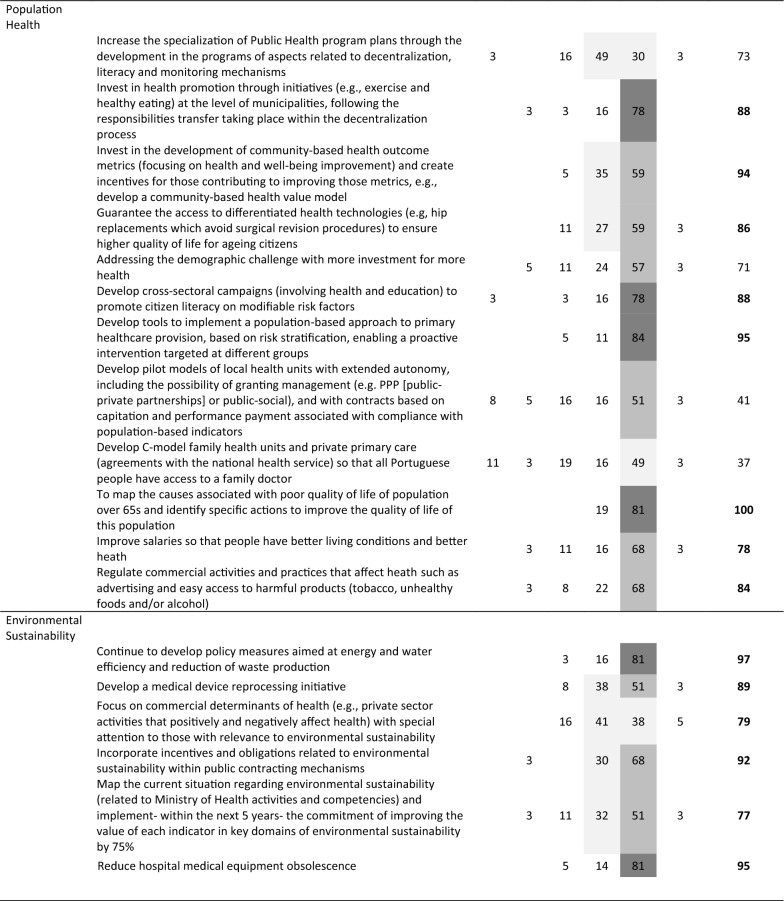
Web-Delphi (second round) results for the recommendations (*N* = 37)

*TD* totally disagree, *D* disagree, *NDA* neither disagree nor agree, *A* agree, *TA* totally agree, *DK/DA* do not know/do not want to answer

Colour scheme:  Net agreement % (Net%) ≥ 25%;  Net% ≥ 50%;  Net% ≥ 75%

Between the Delphi rounds, the average net agreement increased by 4% (with net agreement increasing for 58 recommendations and decreasing for 7 recommendations), and the number of recommendations meeting the net agreement threshold increased from 38 to 43. Notably, recommendations exceeding 95% net agreement grew from 4 to 15 between rounds. Only two recommendations—‘create full dedication for healthcare professionals’ (Workforce) and ‘develop pilot models of local health units with extended autonomy (…)’ (Population Health)—saw significant decreases in net agreement (around 10%). Full results for both rounds are available in Supplementary File 4.

Applying the net agreement rules system, 43 out of 69 recommendations (62.3%) met the (indicative) threshold across PHSSR domains: Governance (8, 72.2% of all Governance recommendations), Financing (4, 44.4%), Workforce (5, 62.5%), Medicines and Technology (6, 54.4%), Service Delivery (6, 50.0%), Population Health (8, 66.6%) and Environmental Sustainability (6, 100%). For these highly agreed recommendations, the average net agreement by domain was 89.9% for Governance, 87.3% for Financing, 85.0% for Workforce, 91.5% for Medicines and Technology, 92.8% for Service Delivery, 89.1% for Population Health and 88.2% for Environmental Sustainability. Three recommendations reached full agreement (100%) among Delphi participants: ‘Implement a merit-based appointment system for those assuming management tasks (…)’ (Governance), ‘Ensure proximity by patients in access to hospital medicines through home delivery or access at the nearest pharmacy’ (Medicines and Technology) and ‘To map the causes associated with poor quality of life of population over 65 s (…)’ (Population Health).

**Fig. 3:**
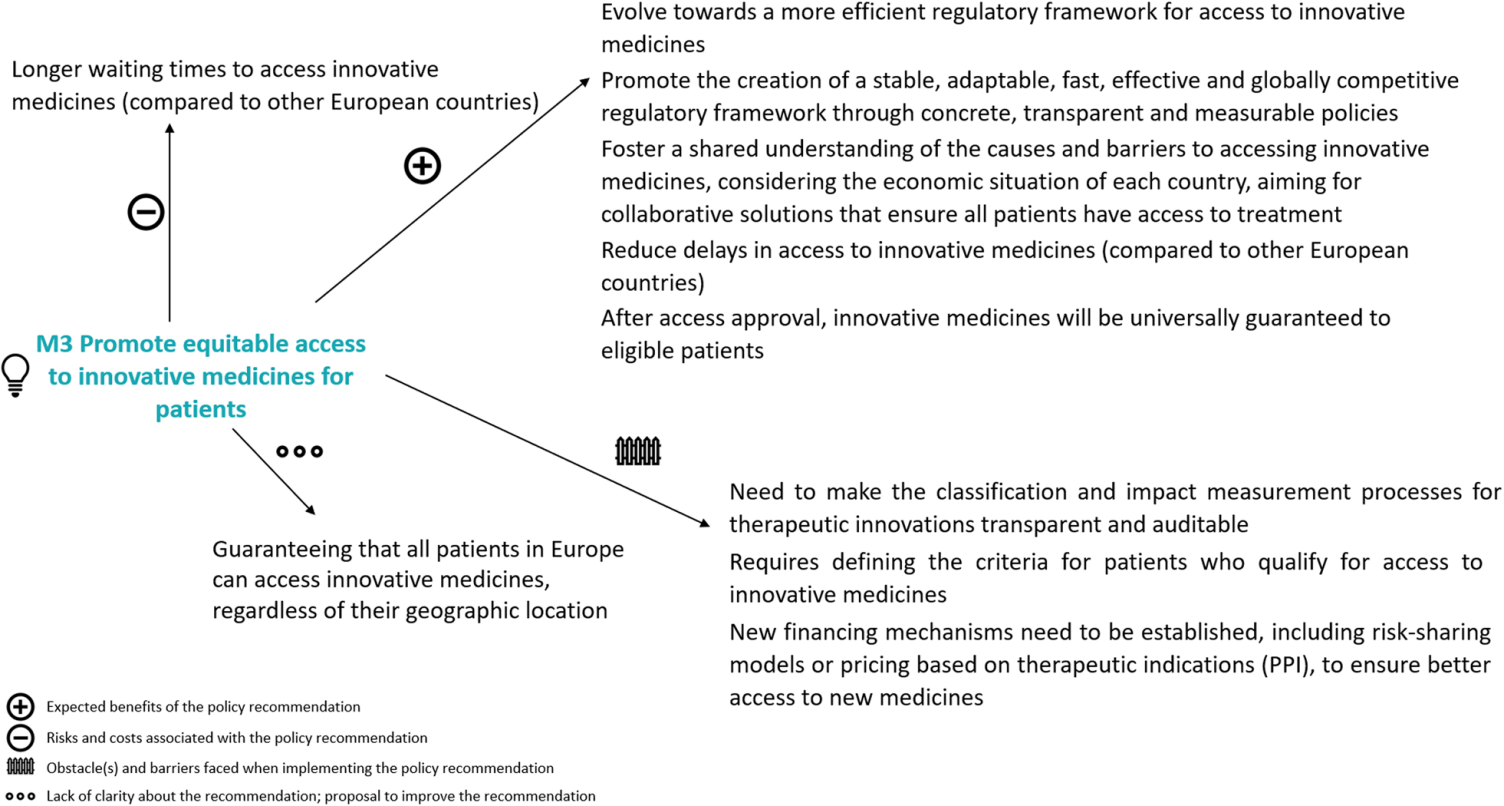
Dialogue map for policy recommendation ‘Promote equitable access to innovative medicines for patients’ (Medicines and Technology PHSSR domain)

A policy dialogue map was developed for each policy recommendation, complementing Web-Delphi results. Figure 3 provides an example for ‘Promote equitable access to innovative medicines for patients’ (in Medicines and Technology, 78.3% net agreement), summarizing key issues and topics raised in stages 2 and 3.

### Stage 4: recommendations’ selection and validation

**Fig. 4:**
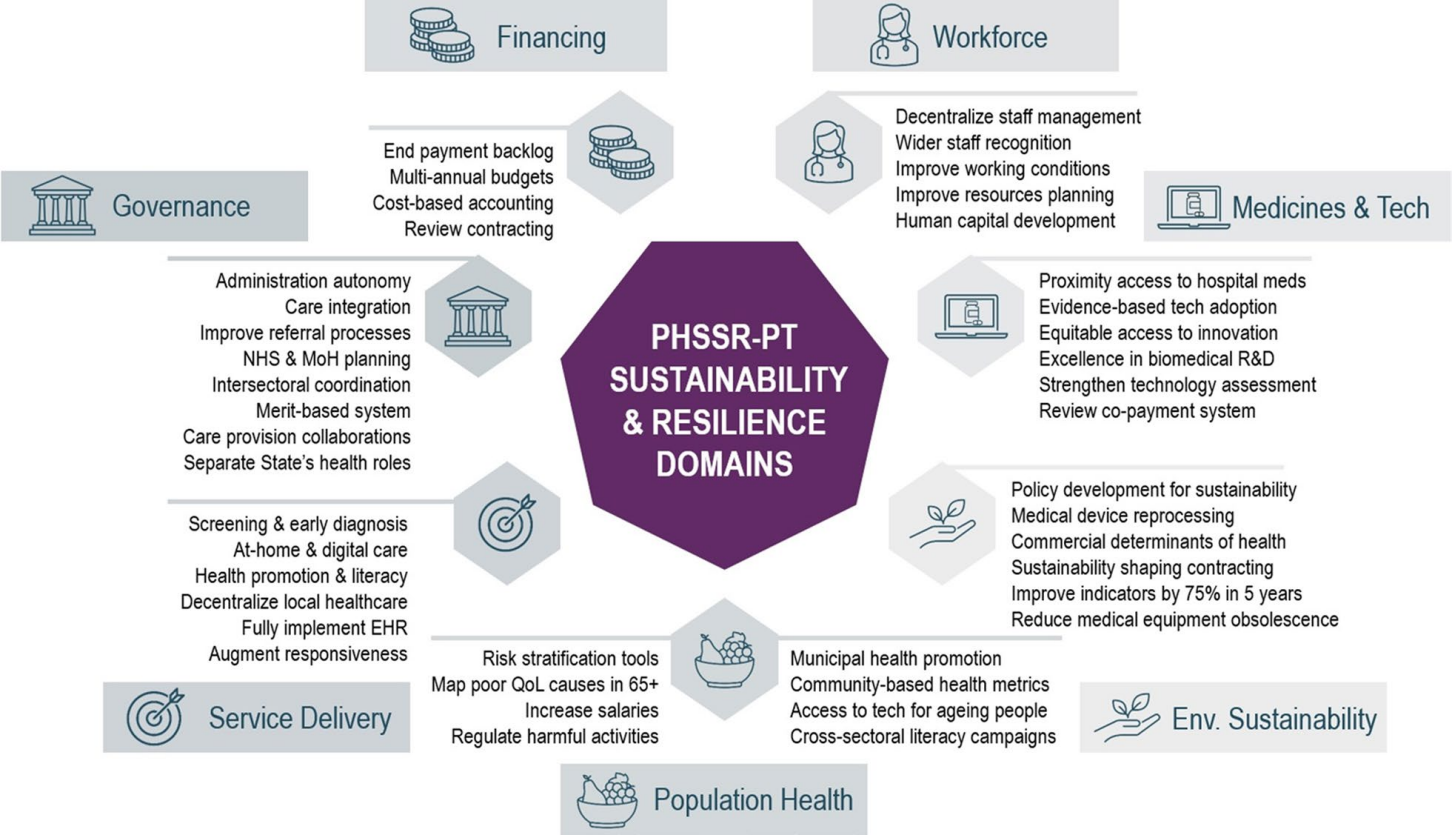
The 43 recommendations emerging from the PHSSR-PT initiative

Members of the core group collaborated in discussing, adjusting and validating the policy recommendations in the final workshop that took place on 6 September 2022 (email interactions allowed participation for those unable to attend the workshop). The group unanimously supported the selection of all 43 recommendations that gathered high agreement in the Web-Delphi. Following the dialogue maps’ analysis for these recommendations and Web-Delphi comments, adjustments were made to the names and descriptions of the selected recommendations. Figure 4 provides an overview of the final recommendations, organized within the seven PHSSR domains. Short recommendation names are provided for concision. Detailed names and descriptions of these selected policy recommendations are available in the PHSSR-PT report [33].

**Fig. 5:**
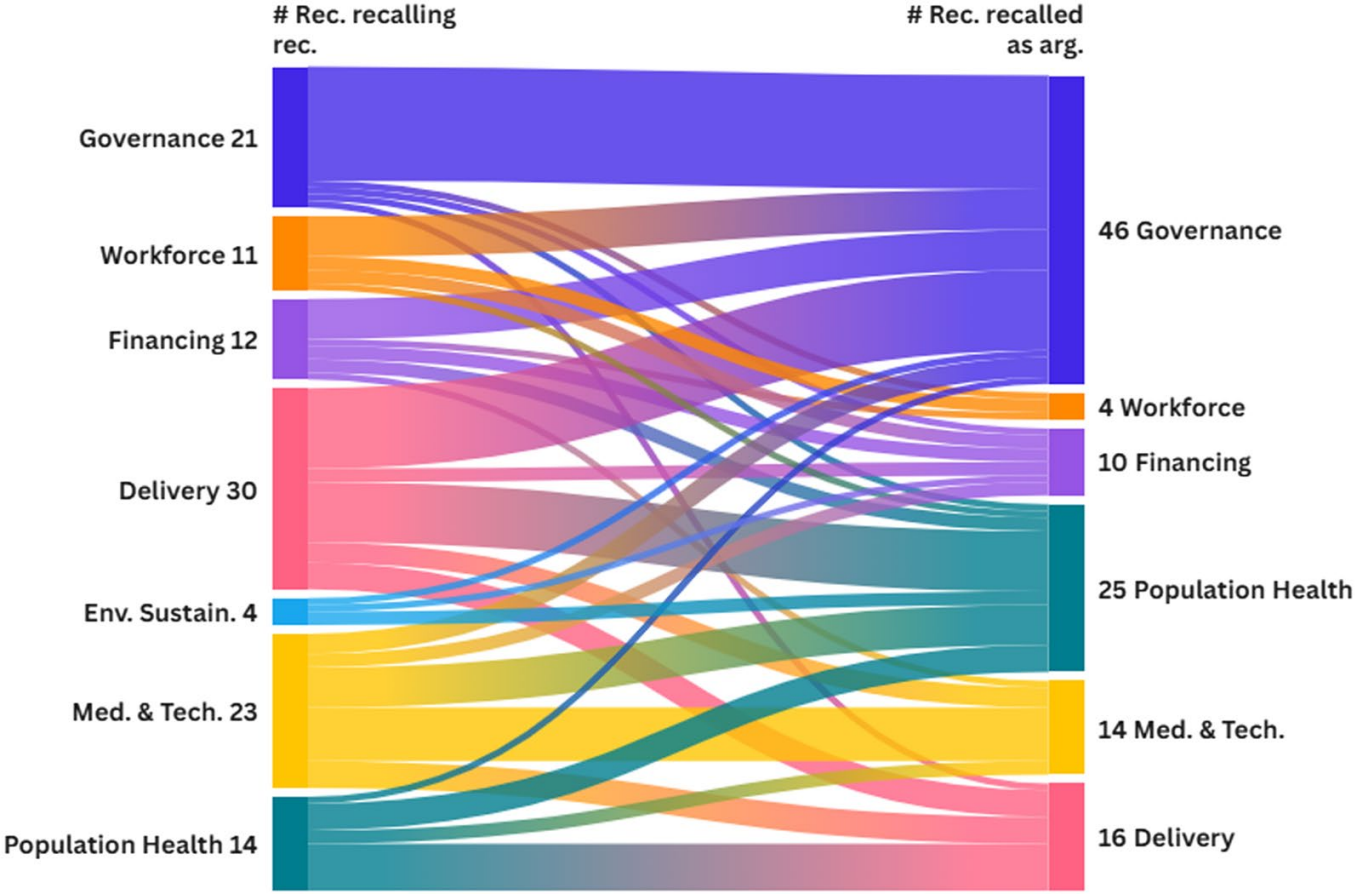
Sankey diagram depicting interconnectedness between policy recommendations shown at the PHSSR domain level

Figure 5 shows the Sankey diagram resulting from applying AIDA, highlighting strong connections between recommendations from the same and across the seven PHSSR domains. On the left, one has the number of recommendations from each domain that recalled other recommendations—for instance, within the rationale and comments of the 8 Governance recommendations, 21 other recommendations were recalled. On the right, one has the total number of recommendations that were recalled in other recommendations—for instance, Governance recommendations were recalled 46 times in recommendations from all the PHSSR domains. One observes in Fig. 4 that Governance, Population Health and Service Delivery recommendations were recalled 46, 25 and 16 times in other recommendations. These areas are thus seen as relevant for the uptake of policy recommendations, given some type of interconnectedness.

### Stage 5: post-assessment

Table 4 presents the results from the post-assessment online survey by members of the core group (completed by eight participants). The average agreement percentages by thematic area were: 100% for the base-case information (statement 1.1), 81.3% for proposed recommendations (statements 2.1–2.4), 87.5% for the Web-Delphi process (statements 3.1 and 3.2), 87.5% for the integrated approach (statements 4.1–4.4) and 72.5% for the PHSSR-PT initiative (statements 5.1–5.5).

**Table 4:** Results from the post-assessment online questionnaire (*N* = 8), presenting group response percentages and agreement (‘agree’ and ‘totally agree’ responses) percentage

Statement	Group responses (%)						Agreement (%)
	TD	D	NDA	A	TA	DK/DA	
1.1 I consider the ‘Fact and Evidence’ sheets were attractive and informative				62.5	37.5		100
2.1 I consider that the adopted procedure for experts to propose recommendations in each domain was clear and allowed an adequate understanding of the task			12.5	50	37.5		87.5
2.2 I consider that the proposed policy recommendations that emerged and were selected in PHSSR Portugal are relevant			12.5	37.5	50		87.5
2.3 I consider that there was an agreement among all experts on the proposed policy recommendations			25	37.5	25	12.5	62.5
2.4 I consider that PHSSR Portugal was a key project in 2022, with a clear contribution to strengthening the discussion on sustainability/resilience in the Portuguese healthcare system		12.5		87.5			87.5
3.1 I consider that the design and language of the Web-Delphi process were accessible and promoted an adequate understanding of the task				62.5	37.5		100
3.2 I consider that the type and quality of available information throughout the Web-Delphi process made it possible to understand the vision/opinion of other participants in the process		12.5	12.5	37.5	37.5		75
4.1 I consider that the facilitation team played an important role in conducting the participatory process, ensuring good communication between participants				50	50		100
4.2 I consider that throughout the participatory process a true constructive process took place between the participants		12.5	12.5	37.5	25	12.5	62.5
4.3 At the end of the participatory process, I had a better understanding of all the questions raised throughout the project				75	12.5	12.5	87.5
4.4 I consider that it was interesting to participate in this project				37.5	62.5		100
5.1 I consider that PHSSR Portugal was a project with opportune timing				50	50		100
5.2 I consider that PHSSR Portugal was the most relevant project in 2022 on the theme of Sustainability and Resilience of the Portuguese healthcare system		12.5	37.5	37.5	12.5		50
5.3 I consider that PHSSR Portugal stood out among the three most relevant projects in 2022 on the theme of sustainability and resilience of the Portuguese healthcare system		12.5		62.5	25		87.5
5.4 I consider that the work developed at PHSSR Portugal was of superior quality		12.5	12.5	62.5	12.5		75
5.5 I believe that PHSSR Portugal will have an impact on the introduction of reforms in the Portuguese healthcare system	12.5		37.5	50			50

*TD* totally disagree, *D* disagree, *NDA* neither disagree nor agree, *A* agree, *TA* totally agree, *DK/DA* don’t know/don’t want to answer

In the survey’s open response questions, respondents suggested that, to implement the recommendations of PHSSR-PT, key initiatives should include: (1) holding discussion forums paired with operationalization documents to thoroughly examine each recommendation; (2) broad disclosure to all stakeholders, coupled with media involvement, ensuring an open discussion environment; (3) prioritizing recommendations and maintaining ongoing dissemination efforts to reflect a commitment from both public and private decision-makers to implement feasible measures; (4) engaging patient groups, persuading majority legislators and securing government support; and (5) organizing panels and round tables to address relevant topics, supported by a comprehensive media campaign, ensuring the direct involvement of influential stakeholders in the decision-making process.

Concerning comments or suggestions about the PHSSR-PT initiative, one respondent considered that the PHSSR-PT initiative was very worthy and timely, although the country’s main problem was not in the diagnosis but in the implementation of the recommendations. Another respondent highlighted that the adopted process was effective in accommodating conflicting views from members of the core group and still led to a wide range of agreed results.

### Communication, public dissemination and discussion and knowledge transfer

The dissemination of the findings from the PHSSR-PT project was conducted through two primary channels. First, three major events were organized: one dedicated to the inaugural launch of the initiative, focusing in disseminating its main objectives and policy recommendations, was held on 7 December 2022, at the Centro Cultural de Belém (Lisbon), and two others focused on public dissemination and discussion of PHSSR results in specific thematic areas, namely access to innovation and clinical research and screening, early diagnosis and treatment and took place on 28 February and 20 June 2023, respectively. These events were organized in partnership with Expresso (the leading weekly Portuguese newspaper) and two took place at the headquarters of Grupo IMPRESA, the media group owning Expresso. The inaugural event received significant media coverage, resulting in over 53 reports across television, as well as national and regional press (a detailed report on media coverage and the calculated return on investment from media activities are available upon reasonable request to the authors). The subsequent thematic events, although smaller in scale, also achieved considerable media attention, particularly highlighting the challenges and opportunities identified. The second dissemination channel involved direct engagement with policymakers and civil society representatives, aimed at public discussion and knowledge transfer. Specifically, meetings were held at the Portuguese Parliament with delegations from the Socialist Party, the Social Democratic Party and the Portuguese Communist Party. Additionally, a meeting was conducted with the President of SEDES (Association for Economic and Social Development), a prominent think tank with recognized contributions to health policy.

## Discussion

We herein discuss the results of the study, from the perspectives of policy insights—covering analysis of sustainability and resilience results for Portugal, framing the results within Portuguese-related and international literature—as well as drawing methodological insights from the policy dialogue. It adds a reflection upon the study limitations.

### Policy insights

#### Sustainability and resilience in the Portuguese health system and policy recommendations

Analysis has shown that the Portuguese NHS is under severe strain in the post-COVID-19, facing major challenges which include staff shortages, underfunding, and unequal access to care. In response, a new policy dialogue produced 43 widely supported recommendations across all PHSSR domains, with key messages being: in the Governance domain, there is a need to promote greater autonomy, coordination, strategy planning and effective partnerships. Within the Financing domain, improving financial control, planning and the availability of cost information emerged as critical. Regarding the Workforce, the recommendations stressed the importance of improving working conditions for health professionals, recognizing merit and making better use of incentive mechanisms. In the field of Medicines and Technology, the focus was on enhancing access, promoting value-based decision-making and supporting innovation. For Service Delivery, the emphasis was on stimulating health promotion and prevention, advancing digital and decentralized care models and fostering partnerships. In terms of Population Health, the recommendations called for actions to foster quality of life and strengthen community-based and targeted interventions. Finally, in the domain of Environmental Sustainability, it was considered essential to address the commercial determinants of health and to promote appropriate incentives and effective management of medical devices and equipment.

Notwithstanding, multiple policy recommendations were found to be interrelated: to be implemented, they require careful planning, engagement and collaboration among stakeholders. Strong connections within and across the seven PHSSR domains can be read in the results of our pragmatic AIDA: with significant intra-domain interconnectedness between Governance and Medicines and Technology recommendations, with Governance, Medicines and Technology and Service Delivery recommendations recalling for many recommendations from other domains and with Governance and Population Health recommendations being recalled by recommendations from other domains. In this context, policymakers should consider potential prerequisites, precedence, spillover effects and synergies among recommendations, rather than approaching them separately. Moreover, practical challenges and costs raise concerns about the multiple requirements for implementing policy recommendations.

Given that multiple recommendations are deemed as consensual, there is a case for developing implementation studies to inform what is required for their implementation. A desirability–doability study [81] could clarify the primary objectives for reforming the Portuguese health system and assess implementation barriers under different scenarios, aiding in prioritization, as suggested by post-assessment survey respondents. Such a comprehensive strategy is essential within the frame of impact assessment, ensuring cohesive health system reform with positive outcomes for sustainability and resilience.

#### PHSSR-PT policy recommendations within Portuguese related literature

Our study produced the first extensive and structured overview of policy recommendations integrating multiple sustainability and resilience perspectives in Portugal. For instance, it includes aspects related to environmental sustainability and population health not included in previous sustainability and resilience studies. In addition, it is the first comprehensive study after the COVID-19 pandemic.

Along the public discussion of PHSSR-PT results, it has been explicitly remarked by several health stakeholders that multiple PHSSR-PT recommendations had been earlier discussed in other forums, being clear that issues regarding the implementation of policies are not new [82] and keep being observed in Portuguese health policy [32]. For instance, the discussion about the implementation of the electronic health record was undertaken elsewhere [83] and concerning aspects regarding funding, professionals’ capacitation, security and interoperability have been priorities identified by the General Directorate of Health (DGS) [84]. In fact, the challenge with the workforce (mainly related to doctors and nurses) has also been under focus recently in a 2023 OECD report [85]. Another report also flags the reduced research and development (R&D) investment in Portugal [20]. PHSSR-PT addresses this issue with recommendations such as ‘Promote equitable access to innovative medicines for patients’ (as found in [83]) and ‘Promote Portugal as a major centre of excellence for biomedical innovation and clinical research’ (Medicines and Technology). The identification of this type of priority concerning the expansion of the R&D capacities was also referred to in DGS report [84]. Willems et al. [30] highlighted the need for valuing primary care practices in reducing health inequalities, the integration of wider staff groups and inter-professional care, improving working conditions, investing in infrastructure and human capital and also reinforcing evidence-based healthcare. All these topics are covered by PHSSR-PT recommendations, particularly within the Workforce and Service Delivery domains. Kuhlmann et al. [31] is highly aligned with [30], taking a greater focus on the workforce aspects of primary care. It advocates for coordinated multi-level Governance (in line with Governance recommendation ‘Optimize communication between primary and secondary care settings by improving referral protocols and mechanisms’), understanding the various existing primary care models and their dynamics (Service Delivery recommendations address such complexity and reinforce the importance of primary care), evidence-based human resources planning and management (aligned with Workforce recommendations for human resources planning and management). Governance reform issues have also been remarked recently [85], highlighting the need to address accountability practices to improve efficiency on spending. Despite the effort for data collection and reporting during COVID-19, PHSSR-PT emphasizes the need to ‘Fully implement electronic health records across the health system’ (Service Delivery) to improve communication between decision-making entities. Socio-economic disparities have been highlighted as key drivers of health inequalities in Portugal [85], being aligned with recommendations such as ‘Improve salaries so that people have better living conditions and better health’ (Population Health).

It is worth noting that several PHSSR-PT recommendations align with those produced in the Gulbenkian Foundation report in 2015 [28]. For instance, both reports highlight as common issues the need to improve governance and coordination within the health system, to implement financial reform, to enhance collaboration between public, private and industry sectors, to increase the role of municipalities, to ensure robust and secure system digitalization, to improve health literacy and education, to expand primary and integrated care models and to promote evidence-based decision-making.

However, PHSSR-PT goes further and provides policy recommendations that can be seen as packages within and across domains. For instance, the Medicines and Technology package includes recommendations towards promoting evidence-based medicine and HTA, towards improving patient access to hospital medicines through home delivery or access at the nearest pharmacy and of innovative medicines, complemented by a review of medicines co-payment system and together with a strategy to promote Portugal as a centre of excellence for biomedical innovation and clinical research. Moreover, few studies have examined health system sustainability and resilience in relation to environmental sustainability. To our knowledge, no previous policy recommendation has proposed committing to improving the value of each indicator in key environmental sustainability domains by 75% within the next 5 years. It emerges clearly from PHSSR-PT recommendations that changes in governance, and focus on population health is required to potentiate and implement recommendations in other domains (as read in Fig. 5).

#### PHSSR-PT in comparison with PHSSR and other international initiatives

While some policy recommendations are highly specific to the Portuguese context, others address gaps and challenges shared by health systems worldwide. The 2023 PHSSR summary report [20] synthesizes findings from 18 distinct country PHSSR initiatives (including Portugal) and provides unified recommendations aligned with (and inspired by) the PHSSR-PT report. These include promoting policy continuity and merit-based appointments, fostering collaboration across care levels and sectors, adopting multi-annual budgets, reviewing (co-)payment systems, improving workforce planning and conditions, investing in education and training, improving HTA processes, strengthening primary care, improving access to medicines, digitally interconnecting care, promoting healthy lifestyles and literacy, enforcing regulations on advertising and access to harmful products, addressing health determinants for vulnerable populations, leveraging technology (e.g. outcome analytics, risk stratification) and advancing environmental sustainability in healthcare (e.g. promoting waste and consumption reduction, obligations in public contracting, medical device reprocessing and reducing equipment obsolescence).

There are also some specific insights that should be taken from the PHSSR global project [20] to Portugal. While Portugal’s centralized approach to pandemic response coordination was viewed as advantageous, the PHSSR global report warns that centralization alone does not ensure success without strong local delivery capacity. This concern aligns with several PHSSR-PT recommendations such as ‘Ensure managerial autonomy of health administrations’ (Governance), ‘Separate the State’s roles in finance, provision, regulation and supervision (…)’ (Governance), ‘Decentralize human resource management (…)’ (Workforce) and ‘Advance local health care delivery (…)’ (Service Delivery). These recommendations are all interconnected as captured by the AIDA analysis.

The interruption of routine screenings and diagnostics, an issue shared with Ireland, Belgium and Canada [20], is tackled in PHSSR-PT through ‘Focus on disease prevention by investing in population-based screening and early diagnosis’ and ‘Develop and strengthen population literacy, health promotion and prevention strategies’ (Service Delivery).

Ultimately, achieving a sustainable and resilient health system in Portugal requires balanced approaches that integrate global best practices and locally tailored solutions.

### Policy dialogue insights

The proposed policy dialogue effectively facilitated a critical assessment of the status quo of the Portuguese health system’s sustainability and resilience across the seven PHSSR domains. It also helped generate high-potential policy recommendations and foster agreement among key Portuguese health stakeholders and experts on those with the greatest potential to enhance the system’s future sustainability and resilience. Partial narrative reports and ‘Fact and Evidence’ sheets were key to setting participants within the same frame and for communication purposes, as confirmed by the post-assessment survey, which acknowledged sheets’ friendliness and informativeness. A core group and an extended group of health stakeholders, engaged through both workshops and Delphi process, agreed to share and discuss their knowledge, further supporting our choice for group-based social processes. This approach was essential, as no single expert could encompass the full range of expertise required across the seven PHSSR domains. As a result, a broad and well-informed set of policy recommendations was collectively agreed upon. The initial stakeholder engagement stage allowed for individual and asynchronous input, enabling stakeholders to freely contribute at their own pace. According to post-assessment results, 87.5% of respondents felt the process was clear and provided a good understanding of the task. During the hybrid workshop, eleven participants reviewed and discussed these proposals, generating 69 recommendations, averaging 6.3 per stakeholder, with four domains having at least 11 proposals each, showing evidence of strong engagement. Recommendations were then assessed by an extended panel of 40 stakeholders in a Web-Delphi process, with stakeholders representing a broad, well-distributed range of professional positions/functions, ensuring diverse perspectives. The high participation rate (78%) and low dropout rate (7.5%) also suggested strong engagement. Post-assessment surveyed participants found the Web-Delphi design and language clear, and 75% felt the information provided was adequate to understand others’ perspectives.

As for the recommendation selection and validation, the core stakeholder group revised policy recommendations based on Web-Delphi results and dialogue maps summarizing process feedback, making only minor changes to names and descriptions. No policies were added or removed, indicating that the Web-Delphi results and net agreement rules were deemed as robust. Forty-three recommendations met the net agreement threshold specified by Eq. 1. Nevertheless, the adopted decision rule was stricter than those commonly found in the literature, such as absolute majority, or a threshold where agreement positions exceed disagreement by at least 75%. Using these alternative criteria would have resulted in selecting 66 (95.7%), 56 (81.2%) or 52 (75.4%) recommendations, respectively. The stringent selection method ensured that only recommendations with very strong agreement and minimal dissent were chosen. Limiting policy proposals avoids relevance or feasibility doubts, confirming study rigour and increasing the likelihood of implementation and impact. It is worth noting that recommendations such as ‘Carry out a structural and organizational reform with the introduction of competition mechanisms for innovation in management’ (Governance), ‘Adopt financing based on value creation and contracting based on results (including health outcomes)’ (Financing), ‘Increase public discussion and disseminate guidelines by the competent authorities to prevent non-grounded treatments in terminal stages of life’ (Medicines and Technology) or ‘Encourage vertical integration of care (primary, hospital, continuing and social), including proximity care’ (Service Delivery) would have been selected under any of the alternative rules systems given Delphi voting results. Additionally, for communication purposes, 43 is a large number of policy recommendations, but the project team considered that after a broad policy dialogue and such levels of agreement there was little room for reducing recommendations within the scope of this study.

Post-assessment survey results confirmed this, with 87.5% of core group members finding the recommendations relevant. Additionally, all stakeholders found the PHSSR-PT project interesting and timely, and 87.5% ranked it among the 2022 top 3 projects in sustainability and resilience for the Portuguese healthcare system.

The designed process was effective in reaching all the study objectives, in terms of producing recommendations, in capturing stakeholders’ attention and in promoting high levels of agreement (while accommodating different and sometimes conflicting views from participants). Nevertheless, although a comprehensive communication and engagement plan was outlined and implemented, some surveyed experts have expressed doubts on whether the facilitated process enabled a genuinely constructive dialogue among participants (which is a common finding in Delphi processes [86]), and half were skeptical about the PHSSR-PT’s ability by itself to promote healthcare reforms in Portugal.

### Study limitations

While this study has several strengths, it is not without limitations. First, the policy dialogue aimed to identify policy recommendations with high potential to improve the sustainability and resilience of the Portuguese health system, without prior insights into which recommendations or groups of recommendations would be most valuable. Potentially, multicriteria value and resource allocation models could be developed to identify the policy recommendations or portfolios offering the highest value for money [53, 87]. This approach would allow for a more detailed evaluation of each recommendation’s benefits, risks, costs and feasibility. Also, AIDA was pragmatically applied in this study, although a complete application of the AIDA method could provide a deeper understanding of interconnected policy recommendations and further work could be done on their requirements.

Second, participatory processes such as workshops, Web-Delphi and surveys have inherent limitations [88, 89]. Cognitive biases could arise from majority opinions or dominant personalities during workshops. The large number of analysed recommendations also might have been overwhelming. To address these, we organized recommendations by PHSSR domain and allowed participants to focus on specific recommendations or opt-out using a ‘Don’t know/don’t want to answer’ option in the Web-Delphi process, as well as make comments. Discussions informed by dialogue maps enabled considering the rationale and comments around the policy recommendations.

Third, it could be argued that the panel of stakeholders engaged in the Web-Delphi panel is not fully balanced. While this is common in Delphi processes due to purposive or convenience sampling, efforts were made to encourage broader engagement, particularly among under-represented groups such as the social sector. However, the distribution aligns with the Portuguese healthcare system, where the NHS accounts for around 60% of health expenditure [90] and approximately for 80% and 59% (in 2018) of inpatient and outpatient spending. Also, it is worth noting that the proposed policy dialogue enabled a wider, structured and more comprehensive engagement of diverse health stakeholders and experts than is common in this type of study.

Finally, while PHSSR-PT addresses sustainability and resilience across the entire Portuguese health system, particular attention was paid to the NHS due to its role in providing universal coverage [91], while there has been a growing role of the private sector provision in recent years. Asking participants also to focus on the private sector could potentially lead to additional policy recommendations.

## Conclusions

This research focused on the sustainability and resilience of the Portuguese health system after the COVID-19 pandemic and on how to reform the system. A novel policy dialogue facilitated open discussion and generated consensus on 43 policy recommendations among leading Portuguese health stakeholders. These recommendations were widely agreed to have significant potential to reform the Portuguese health system and improve its sustainability and resilience.

Implementing these recommendations requires a significant, collective effort from all key stakeholders. A unified plan, grounded in evidence and transparent public dialogue, is essential to prioritize actions and address urgent needs. Coordinated, well-planned efforts across all areas are crucial for effective, lasting reform of the Portuguese health system towards improving its sustainability and resilience. Future work should focus on evaluating the recommendations’ desirability, on assessing their feasibility and costs, and on exploring combinations of policy recommendations within packages.

The structured policy dialogue, which started with scientific evidence, moved to the generation of policy recommendations, structured discussion, and selection of recommendations, while promoting consensus, and engaged a large group of high-level health stakeholders and experts, can be used in many other contexts.

## Supplementary Information

Supplementary Material 1. Template for proposing policy recommendations.

Supplementary Material 2. Fact and Evidence sheets for the seven PHSSR domains.

Supplementary Material 3. Full details and rationale of the 69 recommendations.

Supplementary Material 4. Rounds 1 and 2 voting results.

## Data Availability

The Delphi dataset generated and analysed in this study is available from the corresponding authors on reasonable request (votes and comments from the two rounds). All other data generated or analysed during this study is included in this published article.

## Abbreviations

ADSE: Portuguese Healthcare Insurance for Public Servants
DGS: General Directorate of Health
EHR: Electronic Health Record
HTA: Health Technology Assessment
NHS: National Health Service
OECD: Organisation for Economic Co-operation and Development
PHSSR: Partnership for Health Systems Sustainability and Resilience
PHSSR-PT: Partnership for Health Systems Sustainability and Resilience for Portugal
PPP: Public–Private Partnership
R&D: Research and Development
SINATS: Portuguese Health Technology Assessment System
ULS: Local Health Unit
USF: Family Health Unit
WHO: World Health Organization
