# Supplementary material for: A novel policy dialogue to build sustainable and resilient health systems: findings from PHSSR Portugal
Source: Health Res Policy Syst. 2025 May 19;23:58. doi: 10.1186/s12961-025-01329-5 (PMC12087171; doi:10.1186/s12961-025-01329-5)
Supplement: Supplementary file 1 — Supplementary Material 1. Template for proposing policy recommendations. [file 12961_2025_1329_MOESM1_ESM.pdf]

SUPPLEMENTARY FILE 1: Template for proposing policy recommendations (EN and PT versions).

RECOMMENDATIONS' PROPOSAL FORM

Taking into account the data and evidence provided on the Portuguese health system, propose (for each of the following domains) the policy recommendation that you consider to have the greatest potential to improve sustainability and resilience…

	Recommendation name	Summary justification of the recommendation
…of the **governance** of the Portuguese health system.		
…of the **financing** of the Portuguese health system.		
…of the **provision of care/health care** in the Portuguese health system.		
…of the **human resources** of the Portuguese health system.		
…in the **medicines and health technologies** of the Portuguese health system.		
…of the **population health** of the Portuguese health system.		
…of the **environmental sustainability** of the Portuguese health system.		

Notes:

1. It is not mandatory to suggest recommendations in all domains.
2. It is recommended that this form be completed and sent to the email monica.oliveira@tecnico.ulisboa before the workshop. All recommendations proposed by the experts will be presented at workshop 1.

FORMULÁRIO PARA PREENCHIMENTO DE RECOMENDAÇÕES

Tendo em conta os dados e evidência fornecidos sobre o sistema de saúde em Portugal, proponha (para cada um dos seguintes domínios) a recomendação de política que considere ter maior potencial para melhorar a sustentabilidade e resiliência…

	Nome da recomendação	Justificação sumária da recomendação
…da **governança** do sistema de saúde português.		
…do **financiamento** do sistema de saúde português.		
…da **prestação de cuidados/cuidados de saúde** do sistema de saúde português.		
…dos **recursos humanos** do sistema de saúde português.		
…nos **medicamentos e tecnologias de saúde** do sistema de saúde português.		
…da **saúde populacional** do sistema de saúde português.		
…da **sustentabilidade ambiental** do sistema de saúde português.		

Notas:

1. Não é obrigatório sugerir recomendações em todos os domínios.
2. Recomenda-se que este formulário seja preenchido e enviado para o e-mail monica.oliveira@tecnico.ulisboa antes do workshop. Todas as recomendações propostas pelos peritos serão apresentadas no workshop 1.
