# Supplementary material for: A novel policy dialogue to build sustainable and resilient health systems: findings from PHSSR Portugal
Source: Health Res Policy Syst. 2025 May 19;23:58. doi: 10.1186/s12961-025-01329-5 (PMC12087171; doi:10.1186/s12961-025-01329-5)
Supplement: Supplementary file 2 — Supplementary Material 2. Fact and Evidence sheets for the seven PHSSR domains. [file 12961_2025_1329_MOESM2_ESM.pdf]

SUPPLEMENTARY FILE 2: Fact & Evidence sheets for the seven PHSSR domains.

Partnership for Health System Sustainability and Resilience

Founded by the World Economic Forum, London School of Economics and AstraZeneca

GOVERNANCE

FACT & EVIDENCE SHEET

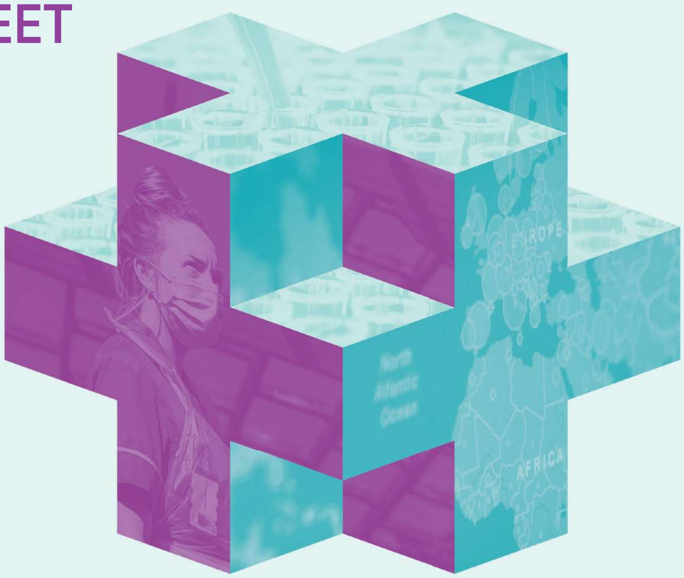

SYNTHESIS OF KEY FINDINGS

DOMAINS	KEY FINDINGS	
	SUSTAINABILITY	RESILIENCE
GOVERNANCE	**Strengths:** clear governance system, mostly centralised and not highly fragmented, which enables decision-making; investments in information systems with potential for improved policy-making	**Strengths:** a command and control system potentiated rapid decision-making along the pandemic; high mobilization and cooperation of institutions during the pandemic; recognition of the role of science, with experts' mobilization and effective daily reporting on the country's pandemic situation
	**Weaknesses:** low accountability in management; underdeveloped policy evaluation systems; fragmented information systems; challenges in implementing health in all and inter-sectorial policies; lack of interconnectedness between independent health institutions	**Weaknesses:** political resoluteness; often reactive rather than proactive pandemic responses; weak contingent planning; still little reflection about learnings and health system changes in the aftermath of the pandemic

KEY EVIDENCE AND DATA

01 Selected features on governance structure and leadership

- National Health Service (NHS) with a centralised command and control structure
- Major responsibilities of the MoH
  - Defining health policy
  - Administrating and managing direct and indirectly multiple institutions (Figure 1)
  - Managing, coordinating, and allocating health state budget resources (Figure 2)
- Main sources of health system funding: government budget (taxes), subsystems, voluntary private health insurance and individual contributions
- Main source of NHS funding: government transfers and subsidies (collected as taxes)
- Main government institutions emanating related laws: MoH, Ministry of Economy and Ministry of Finance
- Key entities with regulatory and supervisory roles: Health Regulatory Agency (HRA), National Authority on Drugs and Health Products (INFARMED), and the Directorate-General for Health (DGH) which assumes:
  - Key responsibilities in health promotion and disease prevention, and in planning and coordinating health care activities and public health programmes
- Key entities in managing and funding the NHS:
  - Central Administration of Health Services (ACSS)
    - managing financial, human and equipment resources
  - Regional Health Administrations (RHAs):
    - implementing national health policy regionally and coordinating all levels of health care
- At the strategic level, there is a National Health Plan (NHP) which:
  - Attempts to promote intersectionality and participation in policies
  - Includes several medium-term targets (issues regarding monitoring)

  BUT

  - Lacks allocation of resources to policies and of mechanisms to enforce the implementation of policies
  - Faces difficulties in adapting the plan to regional and local contexts

02 Selected multi-level governance features

- Some cooperation observed between health institutions and other public sector institutions in multiple areas (e.g. education, social care).

BUT

- Lack of intersectoral institutional structures (e.g. between hospital and primary health care units)
- Lack of interconnectedness between independent health institutions (e.g. National Health Council and Health Regulatory Agency); and limited role of municipalities in the health networks promoting healthy behaviours
- Lack of funding and incentives for collaborative activities
- Little practice of health impact assessments
- Observed issues and challenges related with patient information sharing between entities despite some recent progresses with the implementation of the app MySNS
- RHAs responsible for promoting links between entities under the command and control of the MoH, but lacking instruments and resources to implement those links
- Low level of (vertical) integration of care, with some exceptions, such as local health units, the national network of long-term and integrated care, and coordinated disease-based units

03 Selected accountability, integrity and trust aspects

- Frequent consultation of selected institutional stakeholders within health policy-making
- Several public institutions assume key roles in promoting transparency and accountability on the health policy implementation and execution, for instance:
  - Inspectorate-General of Health-related Activities: responsible for auditing and inspecting health care delivery
  - National Health Council: independent consulting body analysing and advising on health policies
  - Court of Auditors: responsible for external control of public finance and motivates auditing and managerial--related analyses
  - Corruption Prevention Council: is an independent entity that works with the Court of Auditors, developing activities to prevent corruption and related offenses
- Hospitals and public entities have plans to prevent corruption and related offences
- Evidence pointing for overall satisfaction of citizens with the NHS
- Low levels of managerial accountability within the NHS, in particular, related with budgetary deficits and overspending

BUT

- Little involvement of citizens at local and regional decision-making

04 Evaluation of programmes and policies

- The creation of NHS monitoring platforms (e.g. Monitorização SNS and Transparência SNS) allowed access to a wide range of information and online services, made available by multiple health institutions

BUT

- Lack of use of systematic, structured, and transparent (ex-ante or ex-post) approaches to evaluate polices measures
- Fragmented information systems, which create issues for collecting and monitoring data relevant to inform policy-making
- Lack of available and updated cost counting information and imperfect cost accountability in public hospitals

05 Contingency plans, protocols for crises and epidemiological surveillance and early warning systems

- Existence of:
  - Centre for Public Health Emergencies responsible for the:
    - national response to public health emergencies, although there is little information about its activity
  - National Epidemiological Surveillance System (SINAVE) responsible for:
    - analyzing and disseminating data on communicable diseases, which allowed for the preparation of plans to deal with emergency situations calamities; advances in SINAVE were observed along the pandemic

06 Covid-19

1. Preparedness
   - Creation of BI SINAVE to deal, update and integrate large volumes of data to inform
   - Some policy measures specifically designed taking into account the risk in each region

   BUT

   - Weak contingency response plans for respiratory infections for winter and summer seasons
     - Mostly unified and coordinated answer to the pandemic, which enabled a fast and broad implementation of some response measures (including lockdown measures)

2. Some Responses Actions

   a. Pandemic Spread Management
   - Mostly unified and coordinated answer to the pandemic, which enabled a fast and broad implementation of some response measures (including lockdown measures)
   - Improvement of information systems to produce information for pandemic management
   - Creation of a taskforce with advisory responsibilities
   - Weekly performance indicators were developed to monitor the NHS during the pandemic
   - Strengthening of networks of laboratories and of testing capacity
   - Policies and interventions were adjusted along the pandemic

   BUT

   - Government decision-making tended to be more reactive than proactive

   b. Communication
   - Observed difficulties in effectively communicating public health messages
   - Despite daily reporting and communication of data on epidemiological situation to EDC, WHO, other organizations and general public

   c. Coordination
   - DGH lead monitoring and surveillance
   - Local responses by municipalities were diversified and dependent in the resources available (e.g. Lisbon and Oporto health care institutions acquired medical and protection equipment)
   - Measures have been taken to monitor the contacts and isolate transmission chains
   - Observed changes in the relation between the political power and other social actors, such as municipal social support services
   - Temporary increase in (partial) autonomy of health care institutions

   BUT

   - Difficulties to quantify organizational and managerial gains (coordination between different levels) e.g. National Institute of Health, Dr Ricardo Jorge (INSA) has collaborated with several local, regional and national organizations in the response to risk assessment.
   - Issues related with availability of health related data, such as for the use of data by public institutions and for scientific purposes

   d. Resources Management
   - Policies involving wage compensations, employment maintenance measures and loan facilities to some sectors
   - The majority of people declared to trust the government and the health authority in a poll undertaken early in the pandemic

   e. Learning Experience
   - Lack of evidence about health system learnings with the pandemic and about health system changes in the COVID-19 aftermath

ABOUT THIS FACT & EVIDENCE SHEET

This factsheet has been prepared within the scope of the Partnership for Health System Sustainability and Resilience report for Portugal (PHSSR-PT) and has been prepared by Mónica Oliveira, Aida Isabel Tavares, Ana Vieira and Matilde Pacheco.

Following the collection of key data and evidence related with GOVERNANCE in the Portuguese health system, by following the PHSSR framework, this factsheet aims at helping the group of health stakeholders and experts generating evidence-informed solutions and policy recommendations to strengthen sustainability and resilience. A partial report on GOVERNANCE complements this factsheet.

PHSSR is a partnership between the London School of Economics, the World Economic Forum (WEF) and AstraZeneca established in 2020, with more recent partners including Royal Philips, KPMG, Apollo Hospitals and the Center for Asia-Pacific Resilience. The work on Portugal is led by researchers from Instituto Superior Técnico and from Lisbon School of Economics and Management (Universidade de Lisboa).

Partnership for Health System Sustainability and Resilience

Founded by the World Economic Forum,
London School of Economics and AstraZeneca

HEALTH SYSTEM FINANCING

FACT & EVIDENCE SHEET

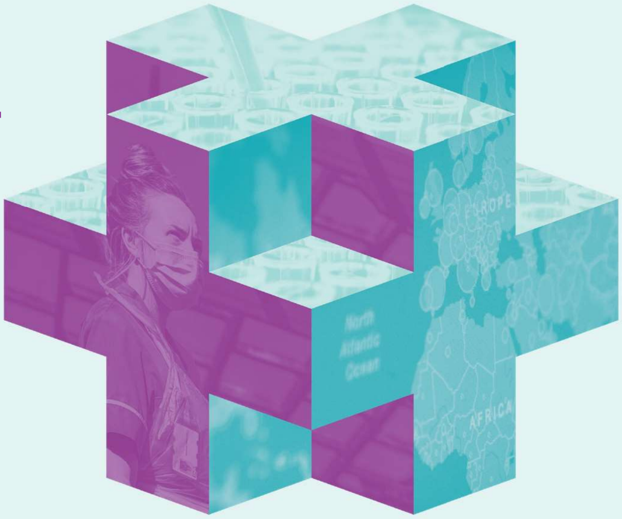

SYNTHESIS OF KEY FINDINGS

DOMAINS	KEY FINDINGS	
	SUSTAINABILITY	RESILIENCE
HEALTH SYSTEM FINANCING	**Strengths:** risk pooling and universal coverage, including care access to immigrants	**Strengths:** new financial policy measures adopted to deal with the pandemic and respond to system financial needs; allocation of PRR funding to improve the NHS
	**Weaknesses:** excessive OOP contributing to inequities in access; systematic budget deficits and debt creation in NHS driving to a kind of retrospective financing; chronic low investment in equipment and professionals; lack of strategic financial planning and diversification in NHS revenue; lack of cost accounting information; excessive average time for paying NHS suppliers	**Weaknesses:** reactive spending under the pandemic; lack of thinking about the need for financial reforms

KEY EVIDENCE AND DATA

01 Key features and trends in financing

- Health system based on a National Health Service (NHS) structure with
    - Funding by taxes (through government budget and then allocated and transferred to the NHS)
    - Having suffered a bailout in 2011 following the adoption of the Economic and Financial Adjustment Programme
    - An increase in out-of-pocket payments that reached almost 31% of current health expenditure in 2019
    - An increasing share of the health budget over the government budget, corresponding to about 7.2% of the GDP in 2020
    - Current expenditure mainly directed to pay:
        - health professionals
        - external services and supplies
        - stocks of pharmaceuticals and clinical material
- NHS revenues are structurally lower than NHS expenditures, generating systematic budget deficits
- NHS debt mounting €1,6M in 2020, with the pandemic appearing in a context of financial fragility of the NHS

02 Healthcare funding-related aspects

- Most of the NHS funding is obtained through the government budget
- The government budget is funded mainly through indirect taxes (about 56%)
    - Some level of regressivity may be arising from health fiscal deductions which benefit higher income families
- Recognised the need for structural (financial-related) policies to address:
    - Increasing age-dependency ratio
    - Declining fertility rate
    - Expected increased public expenditure on health and pensions
- Positive facts:
    - Downward trend was observed for all types of debt since 2018 due to capital injections equal to or greater than €500 million
    - Resources allocation strategies starting to incorporate considerations of efficiency
    - Use of activity-and-performance based contracts and prospective types of payments with providers
    - Increase in long-term care expenditures, considering previous low levels for this kind of care
- Negative facts:
    - Lack of updated information concerning the current level of regressivity and its impact on access to health outcomes
    - NHS Budget is overspent each year and NHS annual deficits create debt
    - Too long average time for paying NHS suppliers (ranging from 140 (in 2017) to 95 (in 2020) days
    - Two main risks associated with health expenditure for the future of the public finances are:
        - Fast aging population
        - Increasing costs of providing innovative care

03 NHS coverage and resource allocation

- NHS
  - Provides universal coverage for all residents, national citizens, and migrants
  - Entails gaps in coverage, which may be provided by private sector after public contract with NHS, include:
    - Oral health
    - Physiotherapy
    - Psychology
    - Diagnostic services
    - Renal dialysis
    - Rehabilitation
  - Very high levels of direct payment (OOP), about 30% of the CHE (current health expenditure)
  - Cost-sharing payments (taxas moderadoras) at the use point were applied until 2022, when they were extinguished
  - Nevertheless, these cost-sharing payments were paid by about 40% of the population; 60% were exempted. Reasons for exemption from the payment of user charges previous to 2022 including:
    - Household income insufficiency
    - Chronic diseases
    - Age (under 18)
    - Pregnancy
  - Around 8% of families face catastrophic health expenditures, 6% being from the poorest families
  - Most of the 2021 government budget for the NHS has been allocated to hospital and vertical programs (nationally organised programs)
  - The National Network for Long-term care (RNCCI) represents a too small share of public health expenditure

04 Paying providers

- The MoH allocates funds to the Regional Health Administrations based on a combination of historical expenditure and capitation
- Primary care units are prospectivelly financed based on capitation and performance
- NHS hospitals
  - The prospective contracts are built through a mixed payment system based on a set of predetermined activities
  - Some activities consider prices per patient treated, per diem, per visit, or hospitalization episode calculated based on DRG and adjusted by hospital case-mix
  - Use of a broad range of institutional incentives and penalties
  - Specific funding related to oncology, HIV, and chronic kidney disease
- Negative Facts:
  - Observed systematic deficits in NHS units which forces a kind of retrospective payment component given at the end of each year
  - Lack of financial mechanisms to ensure access and promoting efficiency in care delivery, for instance, to reduce waiting lists and improve patient choice
  - Weak link between financing mechanisms and population health needs
  - Little use of value-based payment models
  - Lack of cost accounting information to inform managers and hospital administrators

05 Covid-19 pandemic

- Preparedness
  - Lack of health care financing projections to analyse alternative epidemics/pandemics future scenarios
  - Lack of specific public funds planned for use during a crisis
- The health system was responsive to:
  - Shortness of health professionals
  - The low number of incentive care beds
  - Difficulties in the supply of individual protections during the first wave
  - Critical financial constrains
  - Adopt measures such as:
    - Approval of the Recovery and Resilience Plan to improve NHS responsiveness (entailing investments in new hospitals and in improving facilities and equipment)
    - Increase in the public health budget
    - Establishment of an exceptional program for:
      - Acquisition of goods and services
      - Hire NHS professionals
      - Reinforce middle management in NHS hospitals through performance incentives and tighter accountability rules
    - Approval of extra investment to reinforce intensive care beds
    - Diminish barriers to access NHS (mainly bureaucratic) for undocumented immigrants
- Learning and Adapting Effects:
  - Observed sharp contraction in the GDP together with adoption of budgetary measures and the use of macroeconomic stabilizers
  - Reactive spending under the pandemic and increase in public health expenditure
  - Observed worsening of the financial situation of the NHS during the pandemic
  - Little change in contingency financing in light of the pandemic

ABOUT THIS FACT & EVIDENCE SHEET

This fact and evidence sheet has been prepared within the scope of the Partnership for Health System Sustainability and Resilience report for Portugal (PHSSR-PT) and has been prepared by Mónica Oliveira, Aida Isabel Tavares, Ana Vieira and Matilde Pacheco.

Following the collection of key data and evidence related to FINANCING in the Portuguese health system, by following the PHSSR framework, this fact and evidence sheet aims at helping the group of health stakeholders and experts generating evidence-informed solutions and policy recommendations to strengthen sustainability and resilience. A partial report on FINANCING complements this fact and evidence sheet.

PHSSR is a partnership between the London School of Economics, the World Economic Forum (WEF) and AstraZeneca established in 2020, with more recent partners including Royal Philips, KPMG, Apollo Hospitals and the Center for Asia-Pacific Resilience. The work on Portugal is led by researchers from Instituto Superior Técnico and from Lisbon School of Economics and Management (Universidade de Lisboa).

Partnership for Health System Sustainability and Resilience

Founded by the World Economic Forum, London School of Economics and AstraZeneca

SERVICE DELIVERY

FACT & EVIDENCE SHEET

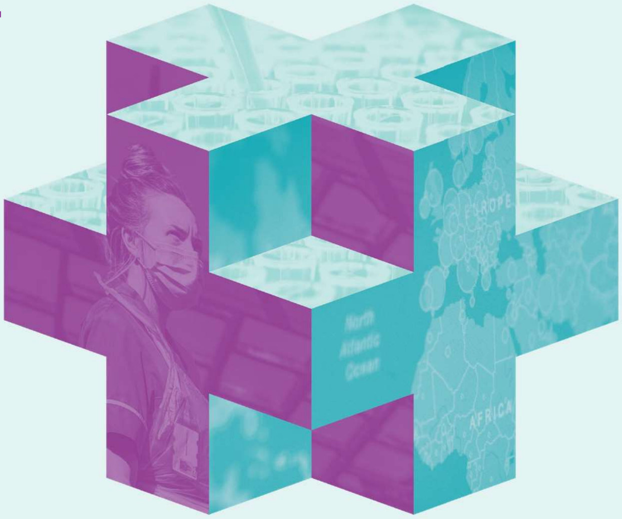

SYNTHESIS OF KEY FINDINGS

DOMAINS	KEY FINDINGS	
	SUSTAINABILITY	RESILIENCE
SERVICE DELIVERY	**Strengths:** extensive primary care network along the territory; investments in the SNS24 phoneline which assumes an increasing importance in the health system; emerging more integrated delivery models and good example of the RNCCI	**Strengths:** sharp increase in telehealth consultations during the pandemic; private sector supply complementary to the NHS and with a flexible supply
	**Weaknesses:** low hospital admission rates, high readmissions, and high length of stay when internationally compared; high waiting lists and times in the NHS; high level of false emergencies, partially reflecting inadequacies in primary care delivery; geographic inequities in health care delivery; still low focus on health promotion and prevention	**Weaknesses:** heavy decline of primary care face to face consultations and of first consultations in hospitals during the pandemic; substantial increase in surgical waiting times in the last two years; restricted capacity in acute and ICU neds

KEY EVIDENCE AND DATA

01 Efficiency of care related aspects

- Admission, readmission and utilization:
  - Low admission rates in NHS hospitals when internationally compared
  - Increasing trend in the rate of readmissions (in 2008, about 4.7% of unplanned admissions were readmissions); more readmission rates for heart insufficiency in 2008 than in 2000
  - Hospital discharges rates have been steady at around 110 discharges per 1,000 population (2019 data)
  - Second highest incidence of hospital infections in the EU
- Beds, occupancy and length the stay
  - Relatively low number of beds per 1,000 population (3.5) compared to the EU average (5.3) (2019 data)
  - Substantially lower adult intensive care beds per 100,000 than the OECD average (2019 data)
  - The second country in the EU21 with the highest occupancy rate of curative (acute) care beds (82.1%), implying pressure on beds (2018 data)
  - The average length of stay has increased in the last decade, reaching 9.4 days in 2019 (e.g. compared to 5 days in the Netherlands)
- Investment-related programs
  - Specific programs have been targeting efficiency gains (for example, SNS24 and the Coronary Green Way), as well as MySNS app
  - Investments in the SNS24 phoneline assumed increasing importance in the health system
- Waiting lists and waiting times trends:
  - Higher median waiting times for elective surgery in Portugal than in the OECD countries before the start of the pandemic
  - Decrease in the percentage of patients on the waiting list respecting the maximum waiting times
  - Introduction of the SIGIC waiting list recovery program to give NHS patients on waiting lists above the clinically acceptable waiting times a surgical voucher so as that they may be transferred to other public or private hospitals
  - But ineffectiveness of the SIGIC in tackling waiting lists during the pandemic

02 Quality of care

- Quality is monitored and promoted by several MoH-related institutions, for instance:
  - The Directorate-General for Health regulates policy and quality standards
  - The Health Regulatory Authority audits the quality of providers and adherence to the law
  - The Medical Professional Association is responsible to analyse problems related to the medical practice
  - SiNATS is responsible to provide information on health technologies quality
- The National Strategy for Quality in Health 2015-2020 frames quality policies, defining targets and priorities to improve the quality of organizational and clinical practice
- Some indicators related to the quality of care are included in the contracts of several NHS providers
- Wide primary care network but increasing difficulties in access to specialized care
- High rate of emergency entries, entailing a substantial level of false emergencies
- Some strategies have been put forward to tackle false emergency services, including:
  - privileged access to hospital care after calling to SNS24 phoneline
  - financial incentives within NHS hospital contracts that consider indicators related to the type of emergency episodes
  - ensuring the offer of emergency consultations in primary care providers

03 Coordination of care and new care models

- NHS management is fairly compartmentalized into primary, hospital, and long-term and palliative care
- There are multiple challenges to promote coordination between health organizations and to make health providers collaborating
- Some initiatives were designed to promote and improve care integration:
  - The National Network for Long-term care (RNCCI) (highly composed of not-for-profit providers) combines teams providing long-term care, social support, and palliative care
  - A few local health units are composed of primary and hospital providers
  - Some vertically integrated models operating, including diabetes programs, have been using an innovative organizational model to provide prevention, diagnosis, treatment, and rehabilitation care, and HIV program endorsing collaboration between several health organizations and community-based organizations focused on the early diagnosis of HIV infection and correct and timely referral of diagnosed people for hospital follow-up

04 Distribution and access to health services

- Health care provision in Mainland Portugal is concentrated in main urban areas and cities (Lisbon, Oporto, Coimbra, and other medium-sized cities).
- Shortage of doctors in rural and in inland areas has been contributing to problems in access to health care due to distance and transport
- In July 2021, 1,2M individuals were not attributed to a general practitioner
- Observed larger socio-economic disparities for unmet needs in Portugal, in comparison to many other countries

05 Programs for prevention and for chronic diseases

- Existence of priority programs to promote healthy lifestyles (e.g. tobacco control and prevention) and prevent chronic (e.g. diabetes) and infectious diseases (e.g. HIV/AIDS infection)
  - These programs include fiscal, advertising, preventative, and health promotion policy measures, such as:
    - Indoor smoking was banned in 2007
    - In 2019, advertising of unhealthy food products to children under 16 was restricted
    - Since 1990, cervical cytologies can be undertaken in primary care
- Creation of the National Cancer Plan in 2016 for early diagnosis and treatment
- Existence of a comprehensive national vaccination program for children
  - Extended in 2018 to include meningitis B, rotavirus, and human papillomavirus vaccination coverage
- Chronic conditions are better managed by primary care than in the EU27, which may partly explain the low hospital admissions for asthma, COPD, congestive heart failure, and diabetes

BUT

- Low focus on health promotion and prevention in the Portuguese system
- Lack of resources allocated to intended policy actions within the national health plan and programs
- Challenges in continuously monitoring indicators of the national health plan and programs

06 Covid-19 pandemic

- What changed?
  - Heavy decline of face-to-face primary care medical appointments along with the pandemic
  - Strong increase in non-face-to-face appointments (including a large number of phone calls provided by GPs under the COVID-19 trace program)
- Consequences of these changes:
  - A decline in diabetes (foot exam and retinopathy) and cancer screening programs
  - In January 2021, the Portuguese League against Cancer estimated that more than a thousand cervical, breast, and colorectal cancers were not diagnosed
  - Longer waiting times for elective surgery
  - The number of days waiting for knee replacement more than doubled between 2019 and 2020, reaching 300 days in 2020
  - Emergency attendance declined in 2020 by 28%
- Issues in maintaining services in a crisis:
  - Serious constraints in hospital capacity during the pandemic in terms of:
    - Beds
    - ICU beds
    - Ventilators
  - Restricted capacity in acute care
  - The number of hospitalizations for COVID-19 soared from April 2020, challenging the supply
  - Most NHS hospitals rapidly became overcrowded and, on several occasions, patients were transferred to hospitals in less-affected regions during the peak of the second wave
- To mitigate these effects:
  - NHS hospitals received donations and loans of ventilators to expand ICU capacity from private companies
  - ICU beds were increased by reducing elective surgery beds
- Co-ordination of care during a crisis:
  - Creation of a taskforce for studying and managing COVID-19 related policy actions
  - Implementation of the National Plan for Preparedness and Response to the New Coronavirus Disease and to respond to the crisis
  - Policy actions were taken to:
    - adapt the delivery of primary care
    - reorganize the hospital network (ensuring COVID free and COVID fully dedicated hospitals)
    - involve private hospitals in delivering some non-elective care
- Learning and adaptation
  - Adaptation
    - Rapid expansion of teleconsultation (around 2 million teleconsultations were taking place per month by the end of 2020)

    BUT

    - Teleconsultations were mostly done via phone
    - High lack of infrastructure to deliver telehealth in primary care settings
  - Learning
    - The contracting process will encourage improvements in access to primary care, namely activities related to the:
      - follow-up of COVID-19 patients
      - surveillance of the chronically ill
      - vaccination, screening, early diagnosis, and family planning programs
    - Public investments from Recovery and Resilience Plan are now being made available to improve e-health care from an integrated perspective within the NHS

ABOUT THIS FACT & EVIDENCE SHEET

This fact and evidence sheet has been prepared within the scope of the Partnership for Health System Sustainability and Resilience report for Portugal (PHSSR-PT) and has been prepared by Mónica Oliveira, Aida Isabel Tavares, Ana Vieira and Matilde Pacheco.

Following the collection of key data and evidence related to SERVICE DELIVERY in the Portuguese health system, by following the PHSSR framework, this fact and evidence sheet aims at helping the group of health stakeholders and experts generating evidence-informed solutions and policy recommendations to strengthen sustainability and resilience. A partial report on SERVICE DELIVERY complements this fact and evidence sheet.

PHSSR is a partnership between the London School of Economics, the World Economic Forum (WEF) and AstraZeneca established in 2020, with more recent partners including Royal Philips, KPMG, Apollo Hospitals and the Center for Asia-Pacific Resilience. The work on Portugal is led by researchers from Instituto Superior Técnico and from Lisbon School of Economics and Management (Universidade de Lisboa).

Partnership for Health System Sustainability and Resilience

Founded by the World Economic Forum, London School of Economics and AstraZeneca

WORKFORCE

FACT & EVIDENCE SHEET

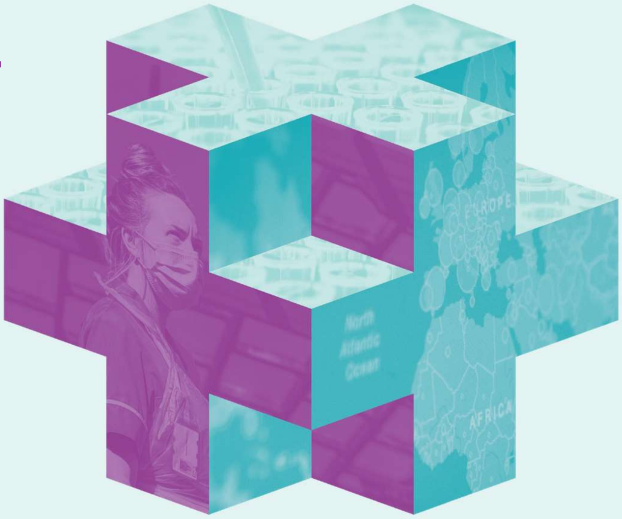

SYNTHESIS OF KEY FINDINGS

DOMAINS	KEY FINDINGS	
	SUSTAINABILITY	RESILIENCE
WORKFORCE	**Strengths:** strengthening of human resources in the NHS following the pandemic	**Strengths:** high level of workforce adaptability during the pandemic, with redeployment and sharing of human resources
	**Weaknesses:** lack of financial incentives to improve workforce productivity in the NHS, with low pay of doctors and nurses; low satisfaction and lack of career development opportunities for doctors and nurses; geographically inequitable distribution of the workforce; lack of workforce strategic planning; workforce tiredness; number of new trained doctors in the NHS does not compensate exits; high shortage of doctors in some medical specialties; instability in medical teams in some contexts due to high use of contracted doctors	**Weaknesses:** no evidence of learning with the pandemic; unsustainable workforce extra costs during the pandemic

KEY EVIDENCE AND DATA

01 Key data on health workforce

- Steadily increasing in the number of doctors and nurses (per 1,000 population) in the last decade
- Higher number of doctors and lower for nurses (per 1,000 population) than the European average, although there is an overestimation of the number of doctors (including doctors with license to practice)
- Increasing number of formal long-term care workers
- Informal care workers with a key role in Portuguese health system (difficulties in having reliable numbers), more relevant than in other European countries, due to:
  - More aged population
  - Low level of long-care expenditure (as a percentage of GDP per capita)

02 Payment and remuneration basic information

- Key information:
  - NHS professionals mostly paid by fixed salary (most civil servants)
  - Doctors' remuneration, on top of fixed salary, receiving pecuniary supplements related to:
    - meal subsidy
    - additional hours
    - emergency hours
    - permanent availability status complement
  - Doctors under a civil servant NHS contract who work 35 hours the values can have a monthly gross salary (no pecuniary supplements) ranging between €1,407- €2,736
  - Exceptionally, GPs working in Family Health Units type B are paid by performance schemes
  - For both nurses and doctors, a substantial amount of salary is obtained by extra-hours and/or by extra work performed in the private sector
  - Legal changes approved in 2016 created incentives for retired doctors to work in the NHS
- Key issues:
  - Low salaries for NHS doctors and nurses compared to European countries salaries and to the Portuguese costs of living
  - Fall in the remuneration of health professionals since 2010 following the bailout and financial crisis have contributed to:
    - Emigration
    - Early retirement
    - Exits from the NHS toward the private sector

03 Workforce training

- Positive facts:
  - Increase in the number of opened vacancies of medical schools in the last decade
  - Observed efforts to increase capacity for specialist training
- Negative facts:
  - Observed bottleneck in the number of vacancies for specialist training
  - Difficulties in filling vacancies for the specialty (related to the high number of dropouts before and during the allocation process)

04 Migration-related findings

- Lack of information and monitoring of health professionals' migration flows
- Increase in the annual foreign-trained doctors' inflow between 2017 and 2018
- Most foreign health professionals working in Portugal are Spanish (36%), Brazilian (26%) and Czech (6%) (2015 reference)
- The main destination for Portuguese doctors is the United Kingdom
- Nurses have been looking to emigrate frequently

05 Key workforce indicators at workplace

- Increasing health workers' absence from the workplace since 2014, especially for nurses
- Lack of doctors in some specialties such as anaesthesiologists, gynaecologists/obstetricians, psychiatrists, internal medicine and GPs
- Unattractive careers in the public sector and better conditions offered by the private sector and abroad
- Containment measures adopted by the MoH significantly impacting NHS workforce
- Recognised need for adopting retention strategies to increase satisfaction and opportunities for career advancement among nurses and doctors

06 Some workforce analysis

- Positive Aspects:
  - About 50% of all Portuguese doctors were GPs in 2018, almost twice as many as the EU27 average
  - Existing teamwork between doctors and nurses in primary care units
- Negative Aspects:
  - Difficulties in defining an adequate workforce strategy, due to the:
    - Issues related with the quality and availability of workforce data
    - Lack of consensus on the future needs of health professionals
    - Little advances to promote staff retention, staff motivation and satisfaction in the NHS (lack of financial incentives to improve workforce productivity in the NHS)
    - More than 400 doctors left the NHS between May and October 2021
  - Number of new trained doctors entering the NHS does not compensate exits
  - Prevailing incentives for civil servants' early retirement
  - High number of NHS professionals moving to the private sector
  - Highly unbalanced distribution of health workers along the territory
  - Limited task shifting
  - Hospital care workforce weaknesses:
    - Lack of studies assessing hospital professionals' satisfaction
    - Use of short-term contracted doctors in some contexts contributing to unstable care teams
  - Primary care workforce weaknesses:
    - Short number of GP
    - Some evidence of burnout in GPs and nurses (more likely to happen among GP than among nurses in primary care)

07 Covid-19 pandemic

- Preparedness
  - Approval of Contingency Plan in March 2020
  - Serious gaps in the workforce (for instance, in internal medicine and in public health specialists)
- Response
  - Some policy measures undertaken at the beginning of the pandemic included:
    - facilitating the hiring of professionals
    - contracting retired health care workers and medical students
    - removing existing caps for payments of extra hours
    - allowing NHS institutions to buy services and hire workers directly for up to four months, as well as to renew short-term contracts
  - More than 10,000 health professionals were hired under exceptional contractual conditions between March 2020 and July 2021
  - Each healthcare unit was responsible to schedule and redeploy human resources according to needs, as well as to provide them with equipment
  - The MoH granted an extraordinary subsidy to NHS professionals working under a contract and directly or indirectly exposed to potentially infected and infected persons
  - Student unions and professional associations helped in establishing banks of volunteers to collaborate in the national response
  - The number of workers answering phone calls at the SNS24 contact centre doubled
  - Implementation of the Cyber-Physical System for Telemedicine and Intensive Care for intensive care treatment of COVID-19 patients
  - High adaptation of health professionals in multiple contexts (e.g. use of telemedicine and distance monitoring for triage and referral of COVID-19 patients)
- Learning and adapting
  - Adapting
    - Strengthening of human resources in the NHS following the pandemic
    - Pharmacists authorized to perform tests and to administer the COVID-19 vaccine and medical students used in health care delivery
    - Unsustainable workforce extra costs during the pandemic
  - Learning
    - In the 2022 legislative elections several parties recognised:
      - Need to improve workforce planning and the careers of the doctors and nurses
      - More autonomy for health institutions to hire professionals
      - Need to ensure that everyone is allocated a GP
    - Uncertainty about whether lessons learned will be put into practice
    - Expected long-term strategy– point to a shift of more resources towards primary and community care, re-deployment of employees to work in different settings and rotate between facilities

ABOUT THIS FACT & EVIDENCE SHEET

This fact and evidence sheet has been prepared within the scope of the Partnership for Health System Sustainability and Resilience report for Portugal (PHSSR-PT) and has been prepared by Mónica Oliveira, Aida Isabel Tavares, Ana Vieira and Matilde Pacheco.

Following the collection of key data and evidence related with WORKFORCE in the Portuguese health system, by following the PHSSR framework, this fact and evidence sheet aims at helping the group of health stakeholders and experts generating evidence-informed solutions and policy recommendations to strengthen sustainability and resilience. A partial report on WORKFORCE complements this fact and evidence sheet.

PHSSR is a partnership between the London School of Economics, the World Economic Forum (WEF) and AstraZeneca established in 2020, with more recent partners including Royal Philips, KPMG, Apollo Hospitals and the Center for Asia-Pacific Resilience. The work on Portugal is led by researchers from Instituto Superior Técnico and from Lisbon School of Economics and Management (Universidade de Lisboa).

Partnership for Health System Sustainability and Resilience

Founded by the World Economic Forum, London School of Economics and AstraZeneca

MEDICINES AND TECHNOLOGY

FACT & EVIDENCE SHEET

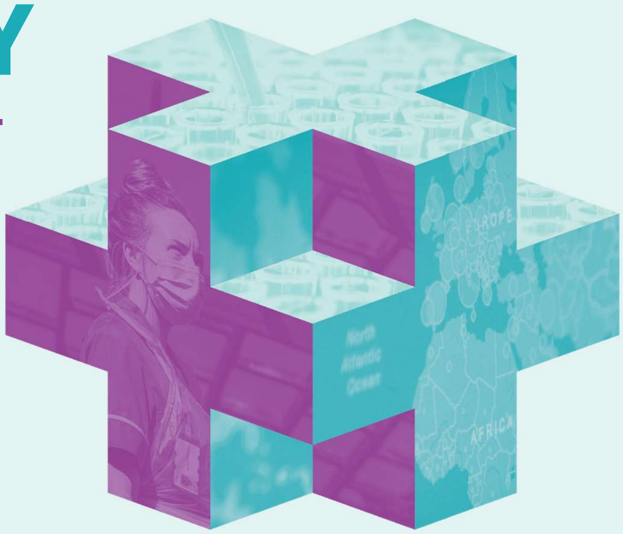

SYNTHESIS OF KEY FINDINGS

DOMAINS	KEY FINDINGS	
	SUSTAINABILITY	RESILIENCE
MEDICINES AND TECHNOLOGY	**Strengths:** new data integration initiatives enabling higher efficiency in care delivery; effective cost containment measures for controlling pharmaceutical expenditure; established economic evaluation procedures	**Strengths:** adaptability and increase in telemedicine use; effective COVID-19 vaccination strategy and delivery
	**Weaknesses:** long time for medicines entry after market authorization; issues in information systems' integration; digital divide; little evaluation of health technologies other than medicine; low level of R&D; financial pressure from hospital medicines	**Weaknesses:** reliance on international markets on COVID-19 related equipment; financial risk related to health technologies' innovation; most telemedicine during COVID-19 based upon phone consultations

KEY EVIDENCE AND DATA

01 Key features related to the adoption of health technologies

- National Authority on Drugs and Health Products (INFARMED):
  - Having strategic competencies regarding health technologies decisions
  - Coordinating the National Health Technology Assessment System (SiNATS)
- SiNATS instituted as:
  - responsible for all matters related to the assessment, pricing, reimbursement, and issuing of recommendations on health technologies
  - consisting of a set of entities and means to assess and reassess all health technologies
  - enabling technical, therapeutic, and economic evaluation of health technologies
  - supported by an information system that collects clinical trial data and makes available information to inform on the quality, economy, efficiency, and effectiveness of the use of technologies
  - including the Commission for Evaluation of Health Technologies (CATS) in charge of:
    - providing opinions and recommendations,
    - analysing economic evaluation studies
    - proposing actions relevant to public health and the adoption of technologies in the NHS
- Regarding the economic evaluation of health technologies
  - Current guidelines for medicines recommend the use of cost-effectiveness analysis (using the EQ-5D-5L instrument) with costs estimated from the perspective of the NHS and complemented by a budget impact analysis
  - Developing initiatives to increase the participation of citizens and patients by INFARMED

  BUT

  - In the case of health technologies, such as medical devices, the guidelines are far less advanced and used relatively infrequently
  - No cost-effectiveness thresholds have been officially recalled
  - Lack of specific procedures to inform de-adoption of low-value medicines decisions
  - Only NHS-related costs are considered (guidelines recommend cost-utility analysis taking NHS perspective)
  - Information systems and mechanisms are less advanced and relatively infrequently used in the assessment of health technologies
- Central purchasing mechanisms in place
  - Applied to a small number of expensive hospital medicines (including HIV/AIDS, C hepatitis, immunomodulators, and oncological drugs)
  - In cases where market authorization is lacking, special financial-based arrangements are negotiated at this level, with some being outcome-based

  BUT

  - Lack of capital funding prospectively set for high-cost new technologies
- About medicines and medical devices spending and related trends
  - Low level of spending per capita on pharmaceuticals in comparison to the EU
  - Increase in medicines spending is mainly due to the growing use of medicines with high reimbursement and the increasing prevalence of chronic diseases (e.g. antidiabetics, anticoagulants) (2019 data)
  - The pharmaceutical network represented 83.5% of the consumer pharmaceutical market (2019 data)
  - Around 10% of pharmacies' sales regard non-prescribed medicines exclusively sold in pharmacies
  - Growth of 10.8% in the sales of reimbursable medical devices (2019 data)
  - Growth in the pharmaceuticals market in terms of volume and value, due to an increase in the value of generics and branded medicines (2019 data)
  - Generics
    - Accounted for almost half of all pharmaceutical sales (by volume) and 63% of the ambulatory market under competition (2020 data)
    - More commonly prescribed in primary care (around 50% generic share), especially concerning the control of chronic diseases

        - Most significant growth, in 2019, has been for lipid modifying agents (an increase of €5.6 million) and analgesics (an increase of €5.3 million)
    - Biosimilars
        - Several policies have been adopted to promote the use of biosimilars; for instance, guidelines for substitution were published and hospital contracting indicators were adopted for this purpose (since 2016)
        - NHS hospital contracts started to integrate performance indicators related to the use of biosimilars in 2017
        - Increasing adoption of hospital biosimilars but still some difficulties are observed in adoption by non-university hospitals
- About the introduction of new drugs and medical devices
    - Observed fluctuations due to:
        - innovation and cost containment pressures
        - regulatory changes in reimbursement
        - shifting consumption patterns
    - Approval of 151 new medicines in 2020, most oncological
    - High approval rate for orphan drugs between 2016-2020
    - New medicines launched in the market accounted for €21 million (five new molecules in the area of diabetes and a new combination of statins both represented more than €10 million (sell-out value) (2019)
    - Since 2015 therapeutic innovation has been prioritized due to the economic impacts on the sustainability of the NHS and the quality of life of patients
    - After market authorization, long stand for medicines becoming available to patients (641 days on average)
    - New Medical Devices Regulation introduced new procedures for equivalent medical devices on the European market and a more judicious benefit-risk assessment

02 Digital health

- Observed multiple advances in the adoption of digital technologies
- Creation of new communication, interoperable channels, and effective e-prescribing solutions for professionals and health care users
- Development of the NHS portal called MySNS and new app Telemonit SNS24
- Several NHS and private pilot projects have been exploring the use of remote patient monitoring within hospital care delivery
- Electronic Health Records (EHR)
    - Used by all primary health care providers and most hospitals
    - Observed some interoperability between different EHR systems
    - Some improvements have been done (Patient, Professional, Institutional, and International Portals on the NHS website)

    BUT

    - Not all information can be linked and accessed
    - Difficulties to follow patients across health care settings
    - Observed differences in platforms across the country
    - Lack of connectedness between hospital data and other data sources, such as disease-based data collections
- Two key programs for digital inclusion and literacy:
    - INCoDE.2030 and "ICT and Society Network" program within the National Strategy for Inclusion and Digital Literacy -ENILD (2015-2020)
- COVID-19 effects on digital health:
    - Selected impacts:
        - Replace face-to-face consultation with teleconsultation through government expanding regulations and payments for physicians and psychotherapists
        - Creation of the social tariff for the provision of broadband internet access for the population with low income or special social needs

        BUT

    - Limited access to face-to-face care
    - 34% of Portuguese people reported some unmet needs in 2020
    - Very high proportion of teleconsultations being phone consultations, with a lack of infrastructure for implementing camera-based teleconsultations

03 Research and development

- Public funded health research is mostly carried out through the Foundation for Science and Technology and public universities
- Positive Findings:
  - High-quality R&D centres and labs focused on infectious and genetic diseases, nutrition and food safety, chronic diseases, environmental and health determinants
  - R&D in health is an area of focus in Portugal
  - Trends:
    - Increase in R&D expenditure
    - Increasing private component of R&D due to qualified employment and technological capacity
    - Increase in the number of clinical trials
- Negative Findings:
  - Low R&D funding for developing health technologies
  - The lack of preparation of the NHS to carry out clinical trials results in lengthy processes and in difficulties in hiring human resources
  - The pharmaceutical industry employed a low number of human resources (2018)
  - High delays in state payments result in high opportunity costs (e.g. in terms of R&D or value creation) and high energy costs for private companies
- MedTech Industry
  - Negative investment levels (the average annual growth rate between 2014-2018 was -14%)

    BUT

  - Presents opportunities, due to its strong specialization in textile products and moulds/plastics
- Smart Health
  - Portugal has been successfully positioned itself as a technology hub in the Smart Health area, which has allowed:
    - Increase in the number of jobs
    - Foreign investments projects tripled

04 Covid-19: Security of supply

- National Medicines Lab is responsible for managing the strategic reserve of medicines and medical devices in disaster situations
- INFARMED has monitored the availability of medicines (including industry stocks) to avoid shortages, with special attention to medicines essential for COVID-19
- Increase in NHS hospital stocks of medicines, medical devices, and personal protection equipment by 20% at the beginning of the pandemic
- Creation of a national (strategic) central reserve
- Centralized procurement of personal protection and testing kits during the first wave, and afterward decentralized to local authorities
- National industry active in producing masks, gloves, gowns, visors, suits, and swabs
- Large amounts of alcohol-gel produced by the National Medicines Lab

05 Covid-19: Vaccination roll-out

- Adoption of a national approach centrally managed and housed in existing health information infrastructure
- Creation of a taskforce (with members from the national defense, internal administration, and health sectors) responsible for:
  - setting the vaccination strategy
  - ensuring logistics
  - ensuring electronic registration of administered vaccines
  - surveillance of adverse reactions
  - promoting a transparent communication
- First investment of around €200 million to acquire more than 22 million doses
- High/ Good organization of the vaccination system
- Effective communication strategy
- Use of alert systems to monitor vaccination and identify any adverse reactions within the National Pharmacovigilance System
- Administration of the third dose of the vaccine in parallel with flu vaccination
- High vaccination rate (surpassed 92% (9 million) of the population with the complete vaccination schedule (2 doses) and has almost 6 million with the booster dose)

06 Covid-19: Advances in digital technologies

Positive advances:

- Shifting face-to-face consultations to alternative digital health solutions both for users and health professionals
- Adoption of teleconsultations by most NHS doctors, mainly phone-based but some professionals using other platforms such as WhatsApp, Zoom, Skype
- Development of several effective health information systems related to COVID infections and vaccinations
- Creation of a self-scheduling portal for vaccination
- Use of the national phone line 2424 to send SMS related to health services (a medical prescription)

BUT

- Ineffective STAYWAY COVID app investment, given technical and data storage protection issues, as well as difficulties to identify contacts in several contexts
- Difficulties related to the delivery of distance-based care (users or caregivers' adaptation, access to those technologies, issues in performing physical examinations, and transmitting or understanding clinical information)
- Lack of capacity by primary care providers to make available teleconsultations using a camera

07 Covid-19: Learning and adaptation

- Several policy medicines and technologies-related actions were taken during the pandemic and may contribute to health system changes:
  - Updating the National Medicines Form
  - Reinforcing the role of the National Commission of Pharmacy and Therapeutics, and enhancing the conditions to address shortage and rupture of medicines
  - Implementing international cooperation measures for information sharing and joint negotiation of innovative medicines
  - Improvements in electronic medicine prescription
  - Development of a strategy to modernize information systems, through the implementation of eHealth in the NHS and development of EHR systems
- Creation of the "Green Light Operation" for the supply of medicines dispensed exclusively in hospitals as a way of guaranteeing the therapeutic continuity of patients who need to go to hospitals to receive their medication

- Need of the NHS to deal with risks related to technological innovation
- Existing financial pressure related to the purchase of hospital pharmaceuticals and clinical materials

ABOUT THIS FACT & EVIDENCE SHEET

This fact and evidence sheet has been prepared within the scope of the Partnership for Health System Sustainability and Resilience report for Portugal (PHSSR-PT) and has been prepared by Mónica Oliveira, Aida Isabel Tavares, Ana Vieira, and Matilde Pacheco.

Following the collection of key data and evidence related to MEDICINES AND TECHNOLOGY in the Portuguese health system, by following the PHSSR framework, this fact and evidence sheet aims at helping the group of health stakeholders and experts generate evidence-informed solutions and policy recommendations to strengthen sustainability and resilience. A partial report on MEDICINES AND TECHNOLOGY complements this fact and evidence sheet.

PHSSR is a partnership between the London School of Economics, the World Economic Forum (WEF), and AstraZeneca established in 2020, with more recent partners including Royal Philips, KPMG, Apollo Hospitals, and the Center for Asia-Pacific Resilience. The work on Portugal is led by researchers from Instituto Superior Técnico and from Lisbon School of Economics and the Management (Universidade de Lisboa).

Partnership for Health System Sustainability and Resilience

Founded by the World Economic Forum,
London School of Economics and AstraZeneca

POPULATION HEALTH

FACT & EVIDENCE SHEET

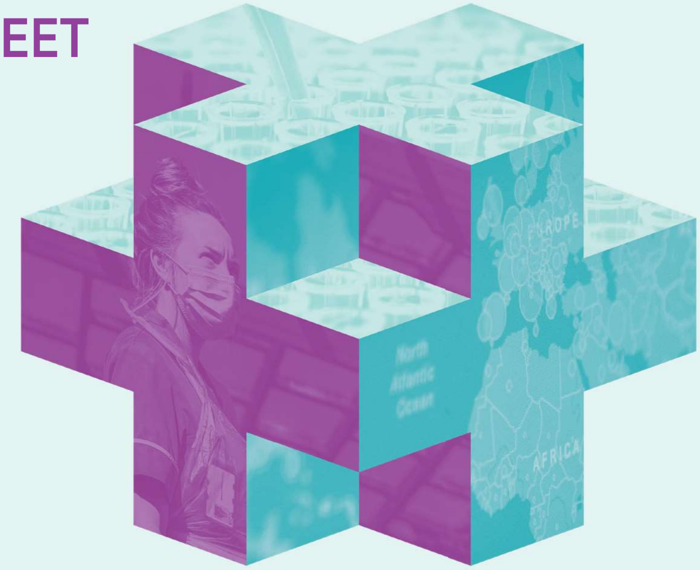

SYNTHESIS OF KEY FINDINGS

DOMAINS	KEY FINDINGS
	**SUSTAINABILITY AND RESILIENCE**
**POPULATION HEALTH**	**Strengths:** good performance in the EU context on mortality and infant mortality rates, and on preventable and treatable mortality; little barriers to access health care (and sometime social protection) by minority groups (including unregistered immigrants); widespread health programs in schools; overall high immunization of the population
	**Weaknesses:** high burden of disease and low quality of life of seniors; high health inequities; high inequalities and inequities in health determinants (and high poverty in the EU context); low health in lifestyle-related indicators (including on overweight and obesity and on physical activity); high incidence of diabetes; high ageing rate and low fertility; COVID-19 pandemic affecting particularly the vulnerable groups; lack of an effective strategy to tackle health inequities

KEY EVIDENCE AND DATA

01 Selected key indicators

POPULATION KEY INDICATORS	PORTUGAL	EU AVERAGE
Relative Poverty	17.2% (2019)	16.5% (2019)
GDP per capita	23,062€ ppp (2020)	29,801€ ppp (2020)
Unemployment Rate	6.9% (2020)	7.1% (2020)
Life Expectancy at Birth	81 years (2021)	80.4 years (2020)
Infant Mortality	2.4/1,000 (2020)	3.3/1,000 (2020)
Treatable Mortality	83/100,000 (2018)	92/100,000 (2018)
Preventable Mortality	138/100,000 (2018)	160/100,000 (2018)
Birth Rate	8.4/1,000 (2019)	9.1/1,000 (2020)
Fertility Rate	1.4% (2019)	1.5% (2019)

Additional Aspects:

- Lower per capita GDP and higher poverty than EU countries
- More aged population than EU countries
- Good performance on mortality-related indicators, but evidence on low self-reported health and quality of life:
  - High morbidity and low quality of life, entailing inequalities, as captured by:
    - Healthy life-years being substantially lower than the OECD average
      - 31% (females) and 43% (males)
    - Higher life expectancy at age of 65 in comparison to OECD average, but entailing gender inequalities
      - 21.5 years (females) and 17.8 years (males)
- Nearly half of the people over 16 reporting a bad or very bad health condition

02 Key evidence on risk factors and inequalities in health

- One third of 2019 death attributed to behavioural risk factors, and poor performance in multiple lifestyle indicators:
  - Observed increase in obesity (nearly a quarter of the population)
  - Overweight (about 20% of teenagers aged 15)
  - High annual alcohol consumption (10.4 l/capita)
  - Diabetes incidence (9.8% of adults) is the second highest in Europe
  - Hypertension (more than 40% of adults)
  - Low physical activity (46% adults) and low vegetable consumption (41.7% adults)
  - Decrease in daily tobacco consumption (14% of adults smoke daily)
  - Pollution (20/ 100,000 died prematurely in 2020).
- Evidence on health and health care related inequalities:
  - Low financial coverage of care translated into high levels of OOP and lower percentages of government spending in different types of care, such as pharmaceuticals and outpatient care
  - Most of the population aged 65 and over report fair, poor or very poor health, with large disparities by income group
  - Self-reporting unmet healthcare needs higher for the population with lower income
  - Energetic poverty affects mainly elderly living in old houses

- Permanent inequalities in the distribution of doctors across the territory
- Food insecurity (affects around 10% of population (in 2015-2016)
- Fast ageing phenomenon (about 25.6% of the population aged 65 or over live in Alentejo)
- Higher age-standardized mortality rate and premature mortality observed in interior regions
- No systematic strategy to tackle health inequalities
- Little attention paid to health socioeconomic determinants

03 Mortality related info

- Main causes of death (2019)
  - cardiovascular diseases (59.3%)
  - malignant tumours (25.5%)
  - respiratory system diseases (10.9%)
  - digestive system diseases (4.3%)
- Substantial incidence of cancer despite being lower than the EU average
- High burden of morbidity and mortality associated with stroke, ischaemic heart disease, pneumonia, and chronic obstructive pulmonary disease (COPD)

AND

- Evidence on lack of actions to reduce behavioral and environmental risk factors associated with leading causes of death

04 Population immunization situation

- Overall high immunization rates for all ages
- Child vaccination rates for diphtheria, tetanus, pertussis and measles (99%) surpassing the (WHO) 95% target

BUT

- Seasonal influenza vaccination for the population over 65 (61%) below the WHO 75% target
- Low vaccination rate for pneumonia (partly justified by lack of information and low economic capacity of the elderly)
- Recognition of the need to develop a specific vaccination plan for the elderly

05 Education and health literacy related programmes in place

- Existence of Education and Health Literacy programmes and policies, which include:
  - The Health Literacy Action Plan (2019-2021) entailing:
    - High levels of health literacy regarding vaccination
    - Medium levels of literacy in health system navigation and in digital health
    - Increase in health literacy levels in recent years
  - A Health Promotion and Education Support Program focusing on:
    - mental health and violence prevention
    - food education and physical activity
    - addictive behaviours and dependencies
    - sexual education
  - A National Integrated Strategy to promote Healthy Food: 2021 legislation established new rules for the preparation of healthier school menus and about the types of food to be sold in schools

06 Selected Covid-19 population health related aspects

NEGATIVE	POSITIVE
• High rate of mortality and infection in comparison with EU countries<br>• Decrease in life expectancy by 0.8 years<br>• Unmet medical needs<br>• COVID-19 particularly affected vulnerable groups such as seniors and migrants<br>• Affected mental health (causing depression, post-traumatic stress disorder and moderate to severe anxiety)	• High adherence to COVID-19 vaccination<br>• Temporary social and health benefits to all immigrants and asylum seekers who applied before 18 March 2020<br>• Approval of a lower co-payment for the pneumonia vaccine for people aged 65 and over

ABOUT THIS FACT & EVIDENCE SHEET

This factsheet has been prepared within the scope of the Partnership for Health System Sustainability and Resilience report for Portugal (PHSSR-PT) and has been prepared by Mónica Oliveira, Aida Isabel Tavares, Ana Vieira and Matilde Pacheco.

Following the collection of key data and evidence related with POPULATION HEALTH in the Portuguese health system, by following the PHSSR framework, this factsheet aims at helping the group of health stakeholders and experts generating evidence-informed solutions and policy recommendations to strengthen sustainability and resilience. A partial report on POPULATION HEALTH complements this factsheet.

PHSSR is a partnership between the London School of Economics, the World Economic Forum (WEF) and AstraZeneca established in 2020, with more recent partners including Royal Philips, KPMG, Apollo Hospitals and the Center for Asia-Pacific Resilience. The work on Portugal is led by researchers from Instituto Superior Técnico and from Lisbon School of Economics and Management (Universidade de Lisboa).

Partnership for Health System Sustainability and Resilience

Founded by the World Economic Forum, London School of Economics and AstraZeneca

ENVIRONMENTAL SUSTAINABILITY

FACT & EVIDENCE SHEET

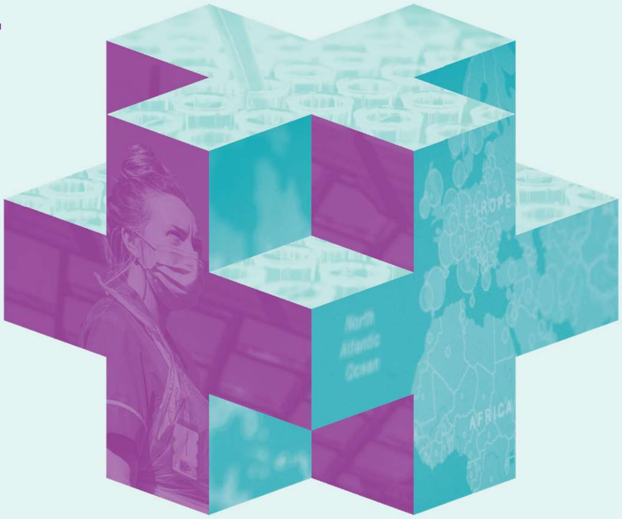

SYNTHESIS OF KEY FINDINGS

DOMAINS	KEY FINDINGS
	SUSTAINABILITY
ENVIRONMENTAL SUSTAINABILITY	**Strengths:** ongoing initiatives and legal frame supporting environmental sustainability in general, and the health system specifically; some initiatives for environmental sustainability lead by the MoH in hospitals reaching maturity
	**Weaknesses:** environmental sustainability measures most centred in hospitals and focused on energy, water and waste; lack of policy actions to implement several strategies related to environmental sustainability; lack of assessment of policies' benefits and costs in the area; scarce research on environmental sustainability in health in the country; lack of incorporation of environmental sustainability in multi-level decision-making in health (e.g. in purchasing equipment, resource allocation)

KEY EVIDENCE AND DATA

01 Key info on environmental costs and benefits of health system activities

- MoH assuming key responsibilities in environmental sustainability
- Two key programs: PEBC (Strategic Plan for Low Carbon) and ECO.AP (Energetic Efficiency Program of the Public Administration), with goal:
  - Reduce greenhouse gases
  - Increase efficiency in the consumption of electricity, water, gas and in the production of waste
- Ongoing Operational Program for Sustainability and Efficiency in the Use of Resources (PO SEUR) entailing
  - Short- and medium-term goals responding to the challenge of changing to a low carbon economy
- Positive findings:
  - Savings in the consumption of electricity, water, gas and in the production of waste in 2018 of approximately €33 Million
  - Actions carried out in some hospitals made it possible to reduce energy up to €40/year and water consumption (80 m^3/month)
  - More transparency in the creation of strategies and information systems that enable monitoring trajectories of efficiency in the use of NHS resources and production due to the introduction of ACSS ranking and quarterly reports
- Negative findings:
  - Environmental related actions are not widespread at the national level
  - Multi-level decisions and resource allocation at the central level has not been considering environmental sustainability dimensions

02 Programs and evidence related with carbon footprint

- Two main programs: PNEC 2030 and RNC 2050 (Roadmap for Carbon Neutrality 2050)
  - Target: achieve carbon neutrality
  - Partly supported financially by investments of the Recovery and Resilience Plan
- Positive aspects:
  - Low level of CO2 emissions per inhabitant (6.2 tons per inhabitant) compared to other European countries (8.1)
- Negatives aspects:
  - Lack of an implementation strategy and unclear information on programs' financing sources
  - Lack of means to calculate the investment required to implement some programs and meet the targets

03 Waste management and measures to reduce its production

- Treatment of waste according to its type in place since 1996
  - Urban waste
  - Non-hazardous hospital waste
  - Biohazard hospital waste (hazardous)
  - Specific hospital waste (hazardous)
- Selected measures taken to reduce the production of hospital waste, according to legal framework:
  - Hospital waste related purchases and service contracts taking into account sustainability criteria
  - Use of waste management plans
- Adopted measures to reduce the production of hospital waste, according to internal management systems, designed to:
  - Minimize the storage of products with short expiration date
  - Enable the use of efficient stock management systems

04 Programs and evidence related to air quality

- Portuguese Environment Agency (APA)
  - Responsible for implementing environment policies, assessing, and managing air quality
- National Air Strategy (ENAR 2020) including as measures:
  - Use of renewable fuels
  - Efficient use of natural resources and raw materials in industrial activity
  - Introduction of emissions' control technologies
  - Use of more efficiency technologies (e.g. renewal of car park)

  BUT

  - Lacking specific policy actions
  - Lacking information on the outcomes to analyse the impact of the implemented measures
- Selected positive findings:
  - Improvements in air quality have been recorded since 1990
  - Local or regional air quality plans in place entail actions for emissions reduction action that are defined according to identified emission sources
- Selected negative findings:
  - Long-term ozone targets are not always achieved in some locations
  - Problems observed in air quality especially in densely populated urban areas (higher concentrations of NO2 and PM109)
  - Issues related to the assessment of the benefits and costs of air quality policies
  - Lack of monitoring of the health effects of air pollution actions

05 Evidence on environmental risks to health

- Identified key vulnerabilities to climate change through:
  - Impact of forest and wildfires
  - High levels of aeroallergens associated with respiratory and cardiac diseases
- Key summer risks
  - Lisbon is the city with the highest health risks during the summer
  - Positive trend of increasing monthly maximum temperature and heat waves
- Key winter risks
  - Low temperatures
  - Higher risk of respiratory infectious in the incidence of infectious diseases (especially seasonal flu) and exacerbation of chronic respiratory and cardiovascular diseases
  - Higher mortality rate
- Extreme weather events-related info:
  - Evidence of negative impact on mental health
  - Six priority risks identified worth of more attention:
    - floods and alterations to coastal zones
    - rising temperatures
    - shortages in public water and energy supplies
    - impacts on natural capital (including land, coastal, marine, and freshwater ecosystems, soils and biodiversity)
    - food production and global distribution chains
    - new pests, diseases and non-native invasive species
- Positive aspects related to environmental risks
  - Creation of two programs "National Climate Change Program" and "National Strategy for Adaptation to Climate Change" to mitigate the climate change effects
  - Creation of the ClimAdaPT.Local project aimed at developing municipal strategies for adaptation to climate change

    - Development of an annual Health Plan for Autumn-Winter
- Negative aspects related to environmental risks
    - Lisbon is the region facing the greatest health risks (related with densely populated urban areas)
    - Lack of analysis of the effects of extreme cold temperatures
    - Lack of specific political actions and associated funding of environmental risks-related programs

ABOUT THIS FACT & EVIDENCE SHEET

This fact and evidence sheet has been prepared within the scope of the Partnership for Health System Sustainability and Resilience report for Portugal (PHSSR-PT) and has been prepared by Mónica Oliveira, Aida Isabel Tavares, Ana Vieira and Matilde Pacheco.

Following the collection of key data and evidence related with ENVIRONMENTAL SUSTAINABILITY in the Portuguese health system, by following the PHSSR framework, this fact and evidence sheet aims at helping the group of health stakeholders and experts generating evidence-informed solutions and policy recommendations to strengthen sustainability and resilience. A partial report on ENVIRONMENTAL SUSTAINABILITY complements this fact and evidence sheet.

PHSSR is a partnership between the London School of Economics, the World Economic Forum (WEF) and AstraZeneca established in 2020, with more recent partners including Royal Philips, KPMG, Apollo Hospitals and the Center for Asia-Pacific Resilience. The work on Portugal is led by researchers from Instituto Superior Técnico and from Lisbon School of Economics and Management (Universidade de Lisboa).
