# Supplementary material for: A novel policy dialogue to build sustainable and resilient health systems: findings from PHSSR Portugal
Source: Health Res Policy Syst. 2025 May 19;23:58. doi: 10.1186/s12961-025-01329-5 (PMC12087171; doi:10.1186/s12961-025-01329-5)
Supplement: Supplementary file 3 — Supplementary Material 3. Full details and rationale of the 69 recommendations. [file 12961_2025_1329_MOESM3_ESM.pdf]

SUPPLEMENTARY FILE 3: Full details and rationale of the 69 recommendations.

Table S3.1: 69 policy recommendations arising from Stage 2 of the policy dialogue.

Domain	Policy recommendation	Summary justification of the recommendation
Governance		
	Ensure the autonomy of the management bodies of health institutions	Captivation by the Ministry of Finance affects the autonomy and management capacity of hospital administrations, causing unpredictability in the fulfilment of obligations. The State should have an attitude of greater trust in public managers and consequent accountability. It is recommended the extinction of captivations in the health area.
	Enable a structure that leads the integration of the provision of care provided at various levels and by different sectors (public, private and social)	Fragmentation and misalignment of health "offers" provide by different health structures and professionals. The new NHS Statute provides the creation of an Executive Directorate that will assume "the coordination of the assistance response of the health units of the NHS, ensuring their functioning in a network". However, there are entities from the private and social sector that provide health care, some of which are contracted/agreed with the NHS. Supervision at that higher level is necessary, so that we have a more efficient health system, avoiding gaps and duplications.
	Develop a decentralized management model for healthcare organizations through the creation of non-executive Board of Directors made up of representatives and responsible for strategic definition, and selection recruitment, and professional management assessment	Government inability to recruit, monitor and evaluate the results of each health management team, adding to the need to develop a transparent model for the selection, recruitment and evaluation of public managers. Development of health institutions in proximity to the community.
	Develop protocols and mechanisms for referral between primary health care and hospitals, in line with the optimization of communication between care	Develop protocols and mechanisms for referral between primary health care and hospitals in order to reduce waiting times and improve the quality of care. Previous research has shown that GPs and family doctors do not always feel confident about the exams to prescribe to their patients before referring them for specialist hospital appointments. The same investigation also showed that specialists doctors consider that patients do not always carry out the necessary exams for the first appointment. This recommendation aims to improve the process of referring patients with gains for the NHS, reduction of waiting times, improvement of the care provided and more satisfaction for patients.
	Carry out a structural and organizational reform with the introduction of competition mechanisms for innovation in management	Revisit the profile of all structures (direct and indirect administration of the State and business sector), review Statutes, attributions, and skills; eliminate contradictions, redundancies, and bureaucratic entropy. Introduce competitive external and internal contractualization mechanisms (Integrated Responsibility Centres, Family Health Units, Public-Private Partnerships). Redistribute competences of Regional Health Administrations by hospitals, groups of Health Centres and Central Administration of Health Systems.
	Extinguish the Regional Health Administrations, replacing them with an executive committee of national health service and universalizing the Local Health Units by the national territory	There is a need to address multiple identified issues:<br>Lack of interconnection and interaction between NHS institutions<br>Lack of autonomy and ability to intervene in a logic of proximity<br>Lack of capacity to integrate other partners from the public, private and social sectors in the public health response.
	Ensure the proper execution of the strategic planning instruments in all hospitals and other institutions of the NHS and the MoH	Ensure proper execution in all hospitals and other institutions of the MoH; ensure adequate use of strategic planning instruments, namely the three-year Plan of Activities and Budget (PAO), the annual Program-Contract (PC) and the management contracts signed with the members of the Board of Directors, in order to guarantee alignment with the priorities of the health policy and provisional management of hospital resources, with autonomy and responsibility for the Board of Directors and for intermediary structures; explain the efficiency and productivity gains that ensure the sustainability of the institutions and lasting reductions arrears.
	Improve intersectoral articulation, in order to be comprehensive and effective, and ensure continuity of care	The definition of a care path that sees the person as a whole, with health and social support needs appears to be an essential goal to improve the quality of life of health users and their caregivers, with undeniable health gains. For this to happen, it is important to improve the articulation that already exists between health and Social Security (for example: RNCCI and, more recently, the recommended articulation, but still very little implemented, within the scope of Informal Caregiver Statute). It is important to ensure good articulation between the various levels of healthcare and

		between these and Social Security, as well as with social responses (ERPIs, Day Centres, Social Support Services) in a perspective of care integration and definition of the care of the health user, ensuring the continuity of different care.
	Appoint for technical competence, with autonomy, responsibility subject to regular audit	To end the political appointments of the directors of the entities providing care, appoint according to competence, evaluate and encourage.
	Promote partnerships and collaborations in the provision of care between the public, private and social sectors in the Portuguese health system	Complementary care and the establishment of partnerships are instruments for the sustainability of the Portuguese health system which includes the public (National Health Service), the private and the social (Misericórdias and Continued Care) sectors, in addition to promoting better health indicators.
	Detach the roles of the State´s funder, provider, regulator, and supervisor, simplifying and empowering its administrative organization	Recommendation considering the following arguments:<br>a) The need to overcome conflicts between the different roles of the State in Health, emphasizing the separation between the funding, regulatory and supervisory State of health care provision and to rationalize the organization, management and functioning of the Health System.<br>b) There is an excessive centralism and bureaucratization of the Public Administration, with difficulty of strategic articulation and coordination; a National Health Service (NHS) too turned on itself, with weak intersectoral relations between health care, public health and Social Security, existence of silos between some organizations and government agencies and disconnection between service providers lacking integrated infrastructures.<br>c) There is a need to create intersectoral dynamics and partnerships between the public, social and private sectors, with increased joint efforts between care providers, development of formal partnerships, including communication networks and collaborative initiatives between Ministries (e.g. Health and Social Security) and the private sector.<br>d) Responding to emerging health needs (home and community care, mid- and long-term inpatient care, palliative care) is insufficient and needs to be reinforced.
Financing		
	End the delay in payments to providers of the National Health Service	Portugal is at the tail of Europe, presenting average deadlines of about 250 days. An approach similar to that adopted by Government of Spain in 2016 is recommended, i.e the creation of an effective payment plan for suppliers (*Plan de Pago a Proveers*) that definitively solved the hospital debts
	Adopt financing based on value creation and contracting based on results (including health outcomes)	Adopt financing based on value creation (value-based healthcare) and on contractualization of results; it is essential to evolve in order to determine community-based outcome metrics (Community Based Outcome Measurements) and within the health system (health outcomes, patient report experience measures and patient report outcome measures), and to create incentives for those who achieve these metrics focused on improving health and well-being of the population
	Adopt multi-annual budgets in the National Health Service	One way to mitigate chronic underfunding of health would be by implementing multi-annual financial management from a medium-term perspective to the detriment of a short-term logic (economic/civil year). In this way, there would be financial stability and continuity of investments, regardless of political cycles or the mandates of the management bodies.
	Extend ADSE to all citizens, only to fund complementary care not covered by the NHS	Extend the ADSE to all citizens, to fund ONLY interventions that are not or are poorly covered by the SNS (mental health, oral health, physiotherapy, etc.)
	Implement a management (or analytics) accounting model	Implement a management, or cost or analytical accounting model, which allows for a more adjusted budget and which can support the contracting of health care;<br>On the other hand, cost measurement makes it possible to support budgeting, measure system efficiency and compare costs between different units, helping decision-making processes and promoting transparency.
	Include, within the scope of financing mechanisms, regulation of agreements and public insurance (e.g. ADSE) adequate and audits	The proposed regulation must be accompanied by a definition of the financing of agreements and audits of the guardianship of these.
	Promoting European convergence of public investment in health by bringing it closer to the average value of EU countries and applying it on the basis of real cost	Converge the value of health investment to the average EU values on the basis of real and correct budgeting, e.g. on the right budget values and appropriate to the levels (of expenditure) and investment without the need for the reinforcement and extraordinary injections of revenue from budgets (in particular hospital budgets);

	budgeting and investment in health	a correct budgeting, and the consequent reinforcement of funds from the health budget, is essential to create better conditions for more efficient management and better planning and predictability in the institutions of the NHS.
	Regulate voluntary insurance and health subsystems as complementary models of financial protection in illness	Regulate voluntary health insurance and health subsystems as complementary models of financial protection in illness given the fragmentation of contributions and inability to guarantee adequate ongoing protection (e.g. definition of a minimum package of services and their duration).
	Review the system for contracting and financing care units in the public, private (agreed) and social (agreed) sectors	Need to review financing system and contractualization, in particular, the indicators that are used, providing autonomy and accountability of managers, as well as to reward those contractualized care units that perform better in health outcomes. Outcome indicators should be given priority to the detriment of process indicators.
Workforce		
	Extend the tasks/competencies performed by the nursing teams (task-shifting)	Allow nurses to accompany chronically ill patients and to prescribe medication.
	Create full dedication for healthcare professionals	Create a body of professionals in the SNS that is more restricted than the current one, but with better remuneration, attractive careers, autonomy and a clear mission.
	Decentralize human resource management within a general career model up-to-date	Development of local career projects for the benefit of the strategic planning of each health care unit and the satisfaction of professionals.
	Expand the concept of human resources of the Portuguese health system beyond physicians and nurses, e.g. including pharmacists, health and diagnostic technicians, clinical secretaries	The health teams are composed of doctors and nurses, but also other health professionals. It is necessary to map these health professionals and their skills/qualifications (scope of practice) in order to identify at what level and by whom the various care can be provided, in the most efficient and safe way (exploring the possibilities of distribution of tasks among all health professionals).
	Educate more specialist doctors in the public and private sector	Increase the number of specialist doctors who start training each year in the public and private sectors
	Improve the working conditions of health professionals through multiple actions	The way of fixing human resources in health in the health system, namely in the National Health Service, has many aspects to implement, such as changing the timetable, increasing flexibility, remuneration based on objectives and performance, time dedicated to research and academic career (if is of interest to the professional), and to improve the conditions of the workplace. All these aspects are essential for the above objective.<br>Today, professionals who work in a hospital cannot provide additional services in that same hospital beyond what is permitted by law, nor can they enjoy the amounts paid to the "laborer" who work side-by-side with them. This restriction forces professionals to work on green receipts in other hospitals, where they do not belong to the professional staff.
	Improve human resources planning in the health sector	Realize the National Inventory of Health Professionals and, with this instrument, create a unit dedicated exclusively to planning and valuing the human capital of the NHS. Improved planning is essential to ensure adequate human resource capacity and adapt the current workforce to new service delivery models of care.
	Valuing human capital through integrated development strategies, contributing to linking talent	Within the Framework of the NHS, it is necessary to distribute adequately and appropriately health professionals, with the development of strategies for recruitment, retention and stability of human resources, with an extension of the remuneration recognition linked to the performance and accountability of teams by results.
Medicines and Technology		
	Adapt and implement SINATS which was approved in 2015 but never implemented	It is necessary to:<br>a) Absolute transparency of the entire evaluation process.<br>b) Avoid the repetition of HTA procedures performed in the EMA at the INFARMED level.<br>(c) Integrate patients' voice into the decision-making process and ensure that the patient perspective is integrated into the definition of outcomes that will be monitored and used for new financing models.<br>(d) remove political power from the decision-making process.<br>e) Make forward-look evaluation and not only retrospective, in order to ensure that technologies that bring lower value in health are not perpetuated in the market.

Increase public discussion and disseminate guidelines by the competent authorities to prevent non-grounded treatments in terminal stages of life	Doctors have information on innovation by the pharmaceutical industry that defends their interests.
Ensure proximity in access to hospital medicines through home delivery or access to the nearest pharmacy	It is important to ensure that every citizen who lacks hospital medicines has access at home or at the nearest pharmacy; The Green Light operation presented itself as a transitory measure, in pandemic time, and should have continued. It was a great added value for the health user with undeniable gains not only in health, avoiding the interruption of therapy due to difficulty in access, but also allowed to save on travel costs and avoid absenteeism at work.
Ensuring that the adoption of technologies is based on evidence-based medicine	From authorization to the use of new medicines and technologies in groups of patients where gains can be observed and where treatments with reduced effectiveness can no longer be used; Promote the use in clinical practice and in the prescription of technologies with recognized cost-effectiveness and abandon alternatives based exclusively on low price that may not be the ones that generate the best results (based on cost-effectiveness).
Strengthen centralized purchasing mechanisms	Centralized purchasing, mainly due to the volume effect and reduction in the duplication of the administrative burden of the acquisition process, allows individual savings to be obtained for hospitals and for the NHS as a whole.
Increase the reimbursement of medicines, prostheses and ocular lenses, and technical aids for families with incomes below 1,5 SSI (social support index)	Reduce catastrophic health expenditure in the most disadvantaged families.
Change the paradigm in public procurement and evolution to value-based purchases	On the one hand, contracting entities should adopt other public procurement procedures other than the usual ones, which favor innovation, whether incremental or disruptive. On the other hand, the repeated practice of the lowest price as the only award criterion has been harmful for patients/users, for health professionals who use medical technologies and for the industry itself. The lowest price as the only reference criterion, leads to many public tenders without competitors. Value-based procurement relates to specific purchasing procedures aimed at improving quality or results and reducing total cost, rather than focusing exclusively on purchasing a specific product at the lowest possible price. This will be a way of encouraging the market to produce value-added products and services.
Promote equitable access to innovative medicines	Evolve into a more efficient regulatory framework on access to innovative medicines. Development and access to innovation has proved to be a time-consuming and bureaucratic process and it is necessary to respond to situations of great inequality in access to new medicines in Europe. The average waiting time between marketing authorisation and patient access varies widely, with differences of more than 700%". While in northern/western Europe, patients' access to innovative medicines takes between 100 and 350 days after marketing authorisation, in southern/eastern Europe the waiting time can reach 850 days. According to the latest European benchmark, EFPIA's W.A.I.T INDICATOR 2020, Portugal is one of the countries with the longest availability time. Portuguese patients are accessing innovative medicines with a delay of more than 4.5 months in relation to an average European citizen, i.e. almost 700 days. As part of the new Pharmaceutical Strategy for Europe, to overcome this situation we need a stable but also adaptable, rapid, effective and globally competitive regulatory structure, resulting in the adoption of concrete, transparent and measurable measures resulting from a partnership between the pharmaceutical industry, the Member States, the institutions of the European Union and, in Portugal, the Government Portuguese. On the other hand, a shared understanding of the causes of delays and barriers to access should be aligned, taking into account the economic conditions of countries, in order to find collaborative solutions to ensure that patients are treated in all European countries with the best therapeutic solutions for their health status, e.g. new approaches and new payment models to finance new medicines.
Promote Portugal as a major centre of excellence for biomedical innovation and clinical research	Promote research and visibility on the return on investment in medicines, with implementation of risk-sharing systems; promote Clinical and Translational Research; clinical trials enable access to medicines free of charge, allow early access to innovation, the production of knowledge essential to the progress of clinical practice, contribute to the strengthening of the qualifications of health professionals and contribute to the improvement of care.
Strengthen health technology assessment (HTA)	Extending HTA to devices and interventions, valuing HTA among the population, and creating a management system for drugs and devices, with data integrated.

	Review the medicines reimbursement system	The system of reimbursement of medicines was evolving incrementally on a general basis, and has many additions and exceptions, in the form of deliberations, dispatches and ordinances, resulting in a fragmented system, which does not promote equity of access (proven by the Portuguese high out-of-pocket in the area of medicines), so needs to be reviewed as a whole.
Service Delivery		
	Investing in disease prevention, investing in population-based screening and early diagnosis	Redirect the focus on prevention and health promotion and not exclusively on disease treatment. Foster the implementation of disease prevention policies, in particular the adoption of healthy lifestyles in order to keep the population as healthy as possible.
	Investing in home health care and digital health associated with this care	Investing in home health care, not only to free up hospital resources, but also because treatment and recovery in the natural "habitat" is more beneficial for patients. For this purpose, there are technological solutions for products and services of remote monitoring that should be leveraged and used.
	Take on the mixed character of the health system, optimising the sustainability of citizens' access to health care in Portugal	To solve problems in the access and quality of care, with long waits and delays in some services and difficulties in the continuity of care (leading to frequent cases of patients "lost" in the system).
	Evaluate Choosing Wisely, the program of wise health choices, to inform future decisions	Evaluate the implementation of Choosing Wisely and identify waste reductions already made; analyze and assess how physicians have already introduced these waste reduction measures into their clinical practice.<br>Reference: The Impact of Choosing Wisely Interventions on Low-ValueMedical Services: A Systematic Review | Milbank Quarterly | Milbank Memorial Fund. .JAMA Health Forum – Health Policy, Health Care Reform, Health Affairs | JAMA Health Forum | JAMA Network.
	Develop and strengthen population education, health promotion and prevention strategies	Taking into account the following rational:<br>a) Strengthen the articulation of the NHS with the private and social sector in order to promote the effectiveness and sustainability of citizens' access to health care in Portugal.<br>(b) This dimension requires a robust and cross-cutting response, only possible through integrated resource management and articulation between the various sectors (public, private, social and cooperative sectors) working in the health area.<br>c) The focus should be on a healthier and more resilient society through health promotion and disease prevention.<br>d) Less than 1% of the health budget is invested in preventing disease which shows that the system is not able to respond to current epidemiological trends.
	Decentralize, increase autonomy and contract	Segment attributions and competencies based on proximity, autonomy and accountability. Increase the capacity to decide and act. Involve local actors in governance.
	Fully implement the electronic health record (EHR) across the health system	Create a single system for the collection, storage and management of users' health information (the RSE) so that, with due authorization, it can be accessed at any point in the health system and in useful time (any health unit, authorized healthcare professionals, any time).<br>The proliferation of different information management systems on users' health without ensuring their interoperability generates a lot of inefficiency, discomfort for the user, a lot of waste, with duplication of exams and consultations and, sometimes, lack of knowledge about essential information for the best provision of care in urgent or emergency situations.
	Encourage vertical integration of care (primary, hospital, continuing, social), including proximity care	In cases where the accumulation of diseases is installed (comorbidity), the integration of care and the management of the chronic disease, often through home care, allow for anticipating exacerbations of the disease and proactively intervening to avoid unnecessary episodes.<br>Priority support for the development of responses, both in the public sector and in the social and private sectors (with policy of incentives), for emerging health care needs, such as home/continuous care, among others, to serve the populations in aging.
	Introduce model C family health units	Increasing the attractiveness of physicians for the public provision of health care, reducing the number of citizens without access to primary health care, and improving the quality of service through the competitive marked created.<br>The C model is characterized by the existence of a contract-program. They can be

		public sector teams or belong to the private, cooperative or social sector. This is an experimental model with a supplementary nature to be regulated by its own diploma.
	Promoting the digital transition as the engine of reform	It is essential to go beyond the transposition of analog procedures into digital format; it is indispensable that information and communication technologies are used to accelerate the creation of health value and generate efficient processes in the provision of care.
	Making primary health care (PHC) more resolute (with greater responsiveness and diversity of services)	"Reform the reform" of PHC, increasing the portfolio of services, and applying performance indicators focused mainly on access.
	Universalizing the model of local health units (ULS) as the basis of the system (including the universalization of family health units (USF) of model B, and investment in (USF) of model C in deprived regions	Universalizing the Local Health Units as the base units of the NHS for territorial organization; universalize the FHU, model B and C, which become the only model for organizing primary health care, investing in the FHU model C in deprived regions. As a note, the Model C FHUs have not been past the paper.<br>The C model is characterized by the existence of a contract-program. They can be public sector teams or belong to the private, cooperative or social sector. This is an experimental model with a supplementary nature to be regulated by it own diploma.
Population Health		
	Increase the specialization of Public Health program plans through the development in the programs of aspects related to decentralization, literacy and monitoring mechanisms	Specialization of the goals and objectives of the National Health Plan to the Local Health Plans, detailing the strategies and measures according to the specificities of communities, territories and individuals.
	Investing in health promotion through initiatives (e.g. exercise and healthy eating) at the level of municipalities, following the transfer within the process of decentralization	Investing on health promotion, through physical exercise and healthy eating programs, for example, allowing to control risk factors, delaying or preventing the onset of diseases that lead to the consumption of health resources and, consequently, imply the sustainability of the system.
	Investing in the development of community-based health outcome metrics (focused on improving the health and well-being of the population) and creating incentives for those who contribute to improving these metrics, that is, developing a community-based health value model	Reducing the burden of disease and investing in a system aimed at improving the health and well-being indicators of the population. The proposed model – the community-based health value model – ensures that public health and health promotion policies become integrated into the health system, with properly measured indicators, and financially incentivized.
	Ensuring access to differentiated technologies to ensure active aging with a better quality of life	Good indicators of life expectancy should be accompanied by a higher quality of life in the population over 65 years. More differentiated medical devices play a crucial role in mobility, and in the treatment of loss of disabling vital senses such as vision and hearing. Examples of this are orthopedic prostheses, intraocular lenses, cochlear implants, pacemakers and coronary stents, and it is essential that the state of the art of these and other technologies are accessible to Portuguese citizens.
	Addressing the demographic challenge with more investment for more health	The recommendation is based on the following reasons:<br>a) Demographic changes and aging have increased some inequalities in access and need to maintain health conditions for chronic diseases.<br>b) Life expectancy in Portugal is at the EU average level, but it has one of the highest rates of old age dependency, combined with less healthy years after age 65 than the EU average.<br>c) In Portugal, in the coming decades there will have to be, necessarily, a greater investment in Health with the older population and this effort will be higher than the average<br>of the European Union (EU).<br>d) This investment will have to consider what resources we aspire, as a society, to allocate to therapeutic innovation
	Develop cross-sectoral campaigns (involving health and education) to promote citizen literacy on modifiable risk factors	From a medium and long-term perspective, the citizen's literacy on how to promote their health and prevent the disease seems essential to not only improve their quality of life, but also to reduce the need to use health services by freeing up means for situations that cannot even be avoided or prevented, related to chronic diseases.

	Develop instruments to implement a population approach to the provision of primary health care, based on risk stratification, that allows proactive intervention, aimed at different groups	The implementation of risk stratification allows the identification of population groups with similar needs. In this way, it is possible to define programs targeted at each group, intervening proactively, to avoid, for example, unplanned admissions. Being able to identify different needs, the allocation of resources can also be more adjusted according to them.
	Develop pilot models of local health units with extended autonomy, including the possibility of granting management (e.g. PPP or public-social), and with contracts based on capitation and performance payment associated with compliance with population-based indicators	Focus of units in population health development, and innovative management models can be implemented through the granting of management to third parties.
	Develop C-model family health units and private primary care (agreements with the national health service) so that all Portuguese people have access to a family doctor	General and family medicine should be the gateway to the Portuguese health system (assuming the role of gatekeeper).<br>The C model is characterized by the existence of a contract-program. They can be public sector teams or belong to the private, cooperative or social sector. This is an experimental model with a supplementary nature to be regulated by its own diploma.
	Map the causes associated with the poor quality of life of the population over 65 years of age and identify specific actions to improve the quality of life of this population	Portugal compares very well in the life expectancy indicator, but very poorly in the quality-of-life indicator after the age of 65. It is necessary to assess the causes of this morbidity, namely avoidable morbidity, which represents an unbearable cost for the country and for families.
	Improving wages so that people have better living conditions and better health	It has been shown that the level of income has an impact on the health of individuals, since it is one of the social determinants in health. We know that the health status of individuals of higher income levels is better and have more longevity. The weak growth of Portuguese wages, without effective real increases in relation to inflation, clearly compromises a positive trajectory of gains in health.
	Regulate business activities and practices that affect health such as advertising and easy access to harmful products (tobacco, unhealthy foods and/or alcohol)	Focusing public health on the commercial determinants of health, negotiating and regulating economic interests whose activity is harmful to the health of the population. The concept of commercial determinants of health refers to "strategies and approaches used by the private sector to promote products and choices that are harmful to health" and covers "factors such as individual behaviors and choices related to consumption and lifestyle, and related to global society, such as consumption risks, political economy and globalization. (https://www.paho.org/pt/noticias/4-1-2022-inaugurada- vitrine-do-conhecimento-sobre-dimensao-comercial-dos-determinantes).
Environmental Sustainability		
	Continue to develop measures aimed at energy efficiency, water and reduction of waste production	Over the last few years, an Environmental Sustainability program has been developed at the Ministry of Health, which began with the Low Carbon Strategic Plan and the Energy Efficiency Program in Public Administration - Health Sector and has now continued with ECO@SAÚDE (Dispatch S.E. SES 10372/2021, of 10/15), which consists of adapting the reality of ECO.AP 2030 (Program for Efficiency of Resources in Public Administration 2030), created by Resolution of the Council of Ministers No. 104/2020, of 24 of November, to the characteristics of the buildings of the Ministry of Health, especially differentiated in the case of hospital entities of the NHS.<br>Its main objectives are to increase the efficiency associated with the consumption of energy resources (electricity and gas), water, and the mitigation of waste production, while simultaneously reducing the emission of gases that enhance the greenhouse effect. In this context, measures are currently being implemented to monitor these consumptions, as well as behavioral measures, either through guides to good practices or through sustainability campaigns that have been developed.<br>A pilot project is also underway for energy efficiency management contracts with ESE, as well as the channeling of community funds to support hospitals to develop energy efficiency actions.<br>That said, to improve the sustainability and resilience of the ENVIRONMENTAL SUSTAINABILITY of the Portuguese health system, it is recommended to continue these measures, as well as:<br>a) Increase in the incorporation of renewable energy sources, in terms of self-consumption, in the building of the Ministry of Health (MS)<br>(b) development of awareness-raising actions at the level of the maximum management bodies of each entity

	c) Concerted strategy at the level of the Ministry of Health, ADENE, DGEG with regard to increasing the capacity to capture european cohesion funds (... and CGEE)<br>d) The incorporation of environmental sustainability in contractualization with hospitals and other health providers.
Develop medical device reprocessing initiative	Waste reduction objective.
Focus on commercial determinants of health with special relevance to environmental sustainability	Focus on public health on the commercial determinants of health, negotiating and regulating economic interests whose activity is harmful to the health of the population. The most obvious example is advertising and easy access to processed foods of animal origin with a strong ecological footprint.
Incorporate in contractualisation mechanisms both incentives and obligations related to the performance of environmental sustainability	Extend the scope of contractualisation mechanisms in view of the energy transition and digital transition objectives; adopt sustained and sustainable practices; opt for alternatives that allow the reduction of emissions; promote the reuse of materials and equipment.
Map the current situation regarding environmental sustainability (related to the work of the Ministry of Health) and implement - in the next 5 years - a commitment to improve indicators in key domains of environmental sustainability by 75%	Environmental sustainability has been outside health policy. By its nature, the provision of health care is a highly polluting activity since a large part of the consumables are for single use and use petroleum derivatives among their raw materials (gloves, masks, uniforms, personal protective equipment, needles, catheters, diapers, etc., etc., etc.). Hospitals, many of them old, are not energy efficient. Much of the waste produced in a hospital requires different forms of treatment due to its organic nature and infectious potential for the community. There are a variety of areas where action is needed to contribute to the environmental sustainability of the planet.
Reduce the obsolescence of hospital medical equipment	It is consensual that the hospital's medical equipment stock is quite deteriorated, with a very high level of obsolescence, which is therefore far below European recommendations (the golden rules).<br>https://www.cocir.org/media-centre/publications/article/cocir- medical-imaging-equipment-age-profile-density-2021- edition.html.<br>In addition to the greater exposure to radiation levels of health professionals and patients, the latest generation equipment is more ecological and environmentally friendly, namely with regard to lower consumption of electricity and water, but also less polluting waste. This recommendation must be accompanied by a mapping of the installed park of equipment, more specifically of the “heavy equipment” (and its implementation requires the use of funds not only from the State Budget, but also from European funds (Horizonte 2030,PRR, etc.).
