# Supplementary material for: A novel policy dialogue to build sustainable and resilient health systems: findings from PHSSR Portugal
Source: Health Res Policy Syst. 2025 May 19;23:58. doi: 10.1186/s12961-025-01329-5 (PMC12087171; doi:10.1186/s12961-025-01329-5)
Supplement: Supplementary file 4 — Supplementary Material 4. Rounds 1 and 2 voting results. [file 12961_2025_1329_MOESM4_ESM.pdf]

SUPPLEMENTARY FILE 4: Rounds 1 and 2 voting results.

Table S4.1. Web-Delphi voting results for the recommendations (first round: N = 40; second round: N=37).

Domain	Policy recommendation	Round 1 Group response (%)						Round 1 Net agreement (%)	Round 2 Group response (%)						Round 2 Net agreement (%)
		TD	D	NDA	A	TA	DK/DA		TD	D	NDA	A	TA	DK/DA	
Governance															
	Ensure managerial autonomy of health institutions			3	28	70		**98**			3	22	76		**98**
	Enable a structure that leads the integration of the provision of care provided at various levels and by different sectors (public, private and social)	3	3	5	18	68	5	74	3	3	5	14	73	3	**75**
	Develop a decentralized management model for healthcare organizations through the creation of a non-executive Board of Directors made up of representatives responsible for strategic definition, selection recruitment, and professional management assessment		18	20	35	23	5	22		19	22	35	22	3	19
	Optimize communication between primary and secondary care settings by developing referral protocols and mechanisms		5	3	25	68		**83**		3	3	14	81		**89**
	Carry out a structural and organizational reform with the introduction of competition mechanisms for innovation in management	5	5	10	35	40	5	55	5	3	5	43	43		70
	Extinguish the Regional Health Administrations, replacing them with an executive committee of national health service and universalizing the Local Health Units by the national territory	13	15	20	20	20	13	-16	14	14	22	24	16	11	-16
	Ensure proper execution of strategic planning instruments in all NHS institutions and the MoH			5	28	63	5	**91**			3	14	84		**98**
	Improve intersectoral articulation, to be comprehensive and effective, and to ensure continuity of care			5	25	65	5	**90**			3	19	78		**97**
	Implement a merit-based appointment system for those assuming management tasks, supported in technical competence and subjected to regular auditing			3	8	85	5	**93**				5	95		**100**
	Promote partnerships and collaborations in healthcare provision involving public, private, and social sectors of the Portuguese health system	3		8	23	60	8	**77**	3		8	8	78	3	**80**
	Separate finance, provision, regulation, and supervision State roles, simplifying and empowering its administrative organization	8		8	23	60	3	67	5		3	30	62		**82**
Financing															
	End the backlog of payments to National Health Service suppliers			5	33	60	3	**93**			3	19	76	3	**95**
	Adopt financing based on value creation and contracting based on results (including health outcomes)	3	8	5	23	60	3	61	3	8	3	16	70		64
	Adopt multi-annual budgets in the National Health Service	3		5	18	70	5	**82**	3		3	19	73	3	**86**
	Extend ADSE to all citizens, only to fund complementary care not covered by the NHS	15	23	28	15	18	3	-43	16	16	32	14	19	3	-31
	Implement a cost-based accounting model			20	28	50	3	**78**			19	19	62		**81**
	Include, within the scope of financing mechanisms, regulation of agreements and public insurance (e.g., ADSE) adequate and audits		5	20	30	38	8	58		5	14	32	46	3	68

Promoting European convergence of public investment in health by bringing it closer to the average value of EU countries and applying it on the basis of real cost budgeting and investment in health	8	5	18	28	43		45	8	5	11	24	51		49
Regulate voluntary insurance and health subsystems as complementary models of financial protection in illness	3	8	20	40	25	5	43	3	5	22	38	27	5	49
Review the contracting and financing system for healthcare providers of the public, private and social sectors			13	38	45	5	**83**			11	41	46	3	**87**
Workforce														
Extend the tasks/competencies performed by the nursing teams (task-shifting)	5	3	13	35	38	8	57	5	3	11	38	35	8	57
Create full dedication for healthcare professionals	10	15	30	20	20	5	-10	11	16	32	19	16	5	-19
Decentralize human resource management within an updated general career model	3		13	43	38	5	**75**	3		8	49	35	5	**78**
Increase recognition and integration of human resources beyond doctors and nurses into health professional teams, e.g., including pharmacists, health and diagnostic technicians, clinical secretaries	3		15	33	45	5	72	3		8	32	51	5	**77**
Training more specialist doctors in the public and private sector		5	25	40	23	8	53		5	19	43	27	5	60
Improve the working conditions of health professionals through multiple actions			5	18	70	8	**88**			5	11	78	5	**89**
Improve human resources planning in the health sector			3	15	78	5	**93**			3	11	81	5	**92**
Value human capital through integrated development strategies, contributing to retaining talent			8	15	70	8	**85**			3	5	84	8	**89**
Medicines and Technology														
Adapt and implement SINATS which was approved in 2015 but never implemented	5	3	10	30	38	15	52	5		8	30	43	14	63
Increase public discussion and disseminate guidelines by the competent authorities to prevent non-grounded treatments in terminal stages of life		5	18	25	45	8	60		5	8	24	57	5	71
Ensure proximity by patients in access to hospital medicines through home delivery or access at the nearest pharmacy		3		30	65	3	**89**				16	84		**100**
Ensure that health technologies adoption is based upon evidence-based medicine			8	23	68	3	**91**			5	14	81		**95**
Strengthen centralized purchasing mechanisms		10	8	38	38	8	56		11		41	38	11	57
Increase the reimbursement of medicines, prostheses and ocular lenses, and technical aids for families with incomes below 1,5 SSI (social support index)	3	3	18	35	38	5	61	3	3	11	32	49	3	69
Change the paradigm in public procurement and evolution to value-based purchases		5	13	40	38	5	68		5	8	43	41	3	74
Promote equitable access to innovative medicines for patients			15	30	48	8	**78**			16	19	59	5	**78**
Promote Portugal as a major center of excellence for biomedical innovation and clinical research			5	18	75	3	**93**			5	8	86		**94**
Strengthen health technology assessment (HTA)			5	38	55	3	**93**			5	30	65		**95**
Review the medicines co-payment system in place			13	48	35	5	**83**			8	49	38	5	**87**

Service Delivery														
Focus on disease prevention, by investing in population-based screening and early diagnosis	3		5	20	73		**87**	3		3	14	81		**89**
Invest in domiciliary care and digital health associated with it		3		30	68		**92**		3		16	81		**91**
Take on the mixed character of the health system, optimizing the sustainability of citizens' access to health care in Portugal	5	8	18	13	50	8	37	5	3	22	11	54	5	49
Evaluate "Choosing Wisely", the program of wise health choices, to inform future decisions	3	3	18	48	20	10	56	3	3	14	57	19	5	64
Develop and strengthen population literacy, health promotion and prevention strategies			5	28	63	5	**91**			3	19	78		**97**
Deepen the local dimension in healthcare delivery through decentralizing, increased autonomy and contracting		3	3	33	58	5	**85**		3	3	24	70		**88**
Fully implement the electronic health record (EHR) across the health system			3	5	88	5	**93**			3	5	92		97
Encourage vertical integration of care (primary, hospital, continuing, social), including proximity care		8	3	20	65	5	69		8	3	11	78		73
Introduce model C family health units	8	5	20	28	33	8	35	11	3	16	27	38	5	37
Promoting the digital transition as the engine of reform		10	3	33	53	3	66		8	3	27	62		73
Make primary healthcare delivery more resolute (with greater responsiveness and diversity of services)			8	25	65	3	**90**			5	22	73		**95**
Universalizing the model of local health units (ULS) as the basis of the system (including the universalization of family health units (USF) of model B, and investment in (USF) of model C in deprived regions	3	10	30	28	28	3	30	3	8	35	22	32		32
Pop. Health and Social Det.														
Increase the specialization of Public Health program plans through the development in the programs of aspects related to decentralization, literacy and monitoring mechanisms	3		18	50	28	3	72	3		16	49	30	3	73
Invest in health promotion through initiatives (e.g., exercise and healthy eating) at the level of municipalities, following the responsibilities transfer taking place within the decentralization process		3	5	20	73		**87**		3	3	16	78		**88**
Invest in the development of community-based health outcome metrics (focusing on health and well-being improvement) and create incentives for those contributing to improving those metrics, e.g., develop a community-based health value model			8	33	58	3	**91**			5	35	59		**94**
Guarantee the access to differentiated health technologies (e.g, hip replacements which avoid surgical revision procedures) to ensure higher quality of life for ageing citizens			10	30	55	5	**85**			11	27	59	3	**86**
Addressing the demographic challenge with more investment for more health		5	13	30	50	3	70		5	11	24	57	3	71
Develop cross-sectoral campaigns (involving health and education) to promote citizen literacy on modifiable risk factors	3		8	23	68		**85**	3		3	16	78		**88**
Develop tools to implement a population-based approach to primary healthcare provision, based on risk stratification, enabling a proactive intervention targeted at different groups			5	23	73		**96**			5	11	84		**95**
Develop pilot models of local health units with extended autonomy, including the possibility of granting management (e.g. PPP or public-social), and with contracts based on capitation and performance payment associated with compliance with population-based indicators	5	3	23	20	48	3	52	8	5	16	16	51	3	41

Develop C-model family health units and private primary care (agreements with the national health service) so that all Portuguese people have access to a family doctor	8	8	20	15	45	5	28	11	3	19	16	49	3	37
To map the causes associated with poor quality of life of population over 65s and identify specific actions to improve the quality of life of this population			3	25	70	3	**95**				19	81		**100**
Improve salaries so that people have better living conditions and better heath		3	10	20	63	5	**77**		3	11	16	68	3	**78**
Regulate commercial activities and practices that affect heath such as advertising and easy access to harmful products (tobacco, unhealthy foods and/or alcohol)		3	13	30	53	3	**77**		3	8	22	68		**84**
Environmental Sustainability														
Continue to develop policy measures aimed at energy and water efficiency and reduction of waste production			3	25	73		**98**			3	16	81		**97**
Develop a medical device reprocessing initiative		3	10	35	48	5	**77**			8	38	51	3	**89**
Focus on commercial determinants of health (e.g., private sector activities that positively and negatively affect health) with special attention to those with relevance to environmental sustainability		3	18	38	38	5	70			16	41	38	5	**79**
Incorporate incentives and obligations related to environmental sustainability within public contracting mechanisms		3		38	58	3	**90**		3		30	68		**92**
Map the current situation regarding environmental sustainability (related to Ministry of Health activities and competencies) and implement- within the next 5 years- the commitment of improving the value of each indicator in key domains of environmental sustainability by 75%		5	10	30	48	8	68		3	11	32	51	3	**77**
Reduce hospital medical equipment obsolescence			8	25	65	3	**90**			5	14	81		**95**

Notes: TD = Totally disagree; D = Disagree; NDA = Neither disagree nor agree; A = Agree; TA = Totally agree; DK/DA = Do not know/do not want to answer.
